# Beyond a Ribosomal RNA Methyltransferase, the Wider Role of MraW in DNA Methylation, Motility and Colonization in *Escherichia coli* O157:H7

**DOI:** 10.3389/fmicb.2019.02520

**Published:** 2019-11-13

**Authors:** Xuefang Xu, Heng Zhang, Ying Huang, Yuan Zhang, Changde Wu, Pengya Gao, Zhongqiu Teng, Xuelian Luo, Xiaojing Peng, Xiaoyuan Wang, Dai Wang, Ji Pu, Hongqing Zhao, Xuancheng Lu, Shuangshuang Lu, Changyun Ye, Yuhui Dong, Ruiting Lan, Jianguo Xu

**Affiliations:** ^1^State Key Laboratory for Infectious Disease Prevention and Control and National Institute for Communicable Diseases Control and Prevention, Chinese Center for Disease Control and Prevention, Changping, China; ^2^Collaborative Innovation Center for Diagnosis and Treatment of Infectious Diseases, Hangzhou, China; ^3^Beijing Synchrotron Radiation Facility, Institute of High Energy Physics, Chinese Academy of Sciences, Beijing, China; ^4^China Institute of Veterinary Drug Control, Haidian, China; ^5^College of Animal Sciences and Veterinary Medicine, Shenyang Agricultural University, Shenyang, China; ^6^Heilongjiang University of Chinese Medicine, Harbin, China; ^7^State Key Laboratory of Molecular Vaccinology and Molecular Diagnostics, School of Public Health, Xiamen University, Xiamen, China; ^8^Laboratory Animal Center, Chinese Center for Disease Control and Prevention, Beijing, China; ^9^School of Biotechnology and Biomolecular Sciences, University of New South Wales, Sydney, NSW, Australia

**Keywords:** *E. coli* O157:H7, MraW, DNA methylation, motility, intestine colonization

## Abstract

MraW is a 16S rRNA methyltransferase and plays a role in the fine-tuning of the ribosomal decoding center. It was recently found to contribute to the virulence of *Staphylococcus aureus*. In this study, we examined the function of MraW in *Escherichia coli* O157:H7 and found that the deletion of *mraW* led to decreased motility, flagellar production and DNA methylation. Whole-genome bisulfite sequencing showed a genome wide decrease of methylation of 336 genes and 219 promoters in the *mraW* mutant including flagellar genes. The methylation level of flagellar genes was confirmed by bisulfite PCR sequencing. Quantitative reverse transcription PCR results indicated that the transcription of these genes was also affected. MraW was furtherly observed to directly bind to the four flagellar gene sequences by electrophoretic mobility shift assay (EMSA). A common flexible motif in differentially methylated regions (DMRs) of promoters and coding regions of the four flagellar genes was identified. Reduced methylation was correlated with altered expression of 21 of the 24 genes tested. DNA methylation activity of MraW was confirmed by DNA methyltransferase activity assay *in vitro* and repressed by DNA methylation inhibitor 5-aza-2′-deoxycytidine (5-aza). In addition, the *mraW* mutant colonized poorer than wild type in mice. We also found that the expression of *mraZ* in the *mraW* mutant was increased confirming the antagonistic effect of *mraW* on *mraZ*. In conclusion, *mraW* was found to be a DNA methylase and have a wide-ranging effect on *E. coli* O157:H7 including motility and virulence *in vivo* via genome wide methylation and *mraZ* antagonism.

## Introduction

*Escherichia coli* O157:H7 is the most commonly isolated enterohaemorrhagic *E. coli* (EHEC) and accounts for more than 90% of clinical EHEC cases (Garg et al., 2005; Friedrich et al., 2007; Xu et al., 2012). It causes diarrheal diseases and other syndromes such as hemorrhagic colitis (HC) and hemolytic uremic syndrome (HUS) with colonization of the intestinal mucosa and subsequent toxin release in the intestinal tract (Friedland et al., 1995). The main virulence factors involved in the intestinal colonization of the host are the type III secretion system (T3SS), curli and flagella (Dobbin et al., 2006; Bretschneider et al., 2007). The regulations of the T3SS and flagella are very complex and affected by many regulators and environmental factors such as pH value, glucose, iron and temperature (Chilcott and Hughes, 2000; Laaberki et al., 2006; Xu et al., 2012; Xu et al., 2013). Recently, it was reported that the utilization of carbon nutrition can affect colonization of *E. coli* in the mouse intestine (Chang et al., 2004; Leatham et al., 2005).

MraW (or named as RsmH) is a 16S RNA methyltransferase (MTase) responsible for N4-methylation of C1402 in bacteria, which is also methylated by another MTase YraL (or named as RsmI) 2′-O-methylation (m^4^Cm) (Kimura and Suzuki, 2010). Recent studies have shown that *mraW* participates in virulence. It was revealed that both *rsmI* and *rsmH* (*mraW*) affected the virulence of *Staphylococcus aureus* in silk worms by contributing resistance to oxidative stress (Kyuma et al., 2015). *mraW* is also likely to contribute to the tolerance to aminoglycoside killing as a *mraW* transposon insertion mutant had a more than 10-fold reduction of gentamicin tolerance in *in vitro* culture (Zou et al., 2018).

DNA methylation has been well studied in eukaryote and is essential in the development and progression of cancer (Furst et al., 2014). It becomes a rapidly growing area of research due to its contribution to improved diagnosis and treatment. Investigating potent and selective small-molecule inhibitors of methyltransferases is important not only for therapeutic intervention but also for understanding the roles of these enzymes in disease progression. Nucleoside analogs such as 5-azacytidine (Vidaza) (Silverman et al., 2002) and 5-aza-2′-deoxycytidine (5-aza) (Decitabine) (Kantarjian et al., 2006) are found as the DNA methyltransferase (DNMT) inhibitors and approved by the FDA for the treatment of myelodysplastic syndromes. These small molecules are incorporated into DNA and thus sequester the activities of the DNMTs (Cai et al., 2016).

However, methylation effect on virulence in bacteria has not been well studied and remains unknown. We hypothesized that methylation in bacteria affect the virulence as it does in eukaryotes. The aim of this study was to determine the effect of *mraW* on virulence in enterohaemorrhagic *E. coli* O157:H7 and the relationship between methylation and virulence.

## Results

### Deletion of *mraW* Led to Reduced Motility and Flagellin Production/Secretion

**FIGURE 1:**
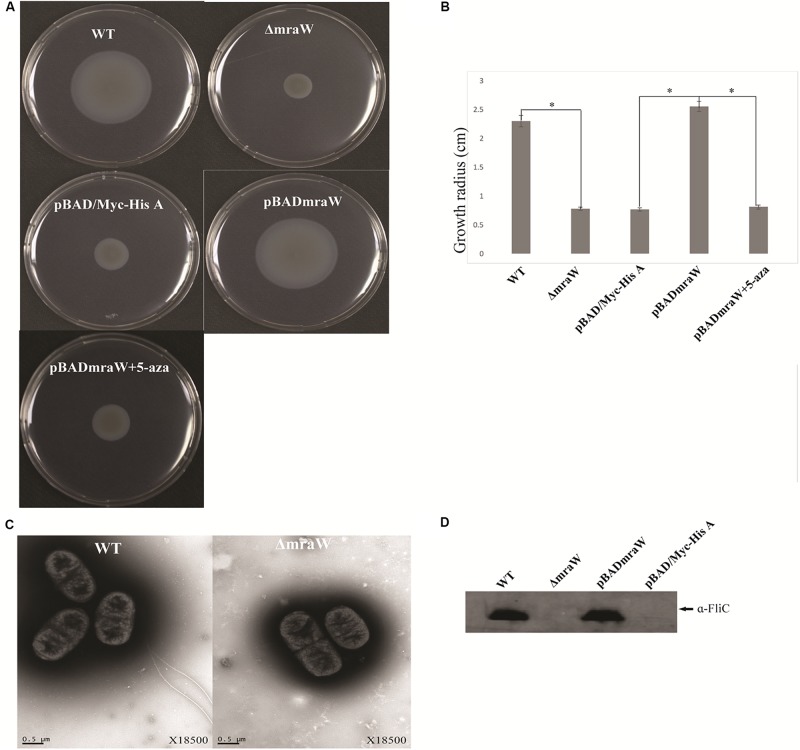
Effects of *mraW* on motility. **(A,B)** Representative images and growth radius of swimming motility for the wild-type EDL933 (WT), the *mraW* deletion mutant (ΔmraW), an empty vector control in the mutant (pBAD/Myc-HisA), complemented strain (pBADmraW) and complemented strain with 5-aza (pBADmraW+5aza). pBAD is an empty vector control. Error bar shows the standard deviation from three independent experiments. Differences were analyzed for significance by using *t*-test. Significant difference between two strains (*P* < 0.01) isindicated by a ^∗^ with a linked line. **(C)** Transmission electron micrographs of wild-type EDL933 and the *mraW* mutant (scale bar, 0.5 μm). **(D)** Immunoblot analysis of FliC protein in the whole cell lysates prepared from wild-type EDL933 (WT), the *mraW* deletion mutant (ΔmraW), empty vector control strain (pBAD/Myc-HisA) and complemented strain (pBADmraW) grown in LB. Arrows indicate a reactive band corresponding to FliC detected with anti-H7 FliC antibodies.

The gene encoding MraW was deleted in *E. coli* O157:H7 strain EDL933 resulting in a mutant strain designated as EDL933ΔmraW. The motility of the wild type and the mutant was determined by the radius of chemotactic ring which was 2.3 ± 0.1 cm and 0.783 ± 0.03 cm, respectively. The difference is statistically significant (*t*-test, *P* < 0.01) (Figures 1A,B). The decrease in motility in the *mraW* mutant was also correlated with a decrease in the production or secretion of FliC as determined by western blotting using anti-H7 antisera (Figure 1C). To determine whether the *mraW* deletion can be complemented, we created pBADmraW, a low copy number plasmid carrying *mraW* which was transformed into EDL933ΔmraW. The radius of the chemotactic ring of the complemented strain (EDL933ΔmraW+pBADmraW) was 2.55 ± 0.09 cm (Figure 1A), which is similar to the wild type. Thus, the decrease in motility of the mutant was almost complemented back by the plasmid pBADmraW expressing *mraW*. The generation time of EDL933, EDL933ΔmraW and EDL933ΔmraW+pBADmraW was 35.7 ± 0.06 min, 35.9 ± 0.04 min and 35.8 ± 0.1 min, respectively. Hence the difference in motility and production of FliC was not due to the growth rate which was similar among the mutant, the complemented strain and the wild type.

To investigate whether the reduced motility of the EDL933mraW mutant was due to decrease of surface flagella, bacteria were inspected by transmission electron microscopy (TEM) to visualize surface flagella at a magnification of 9,700×. Thirty fields of view were randomly selected and about 50–200 cells counted. The majority (95%) of the *mraW* deletion mutants were found to have no flagella, with the remaining 5% of the cells having one to two flagella (Figure 1D). In contrast, most of the cells from the wild-type EDL933 had surface flagella although the number of surface flagella was limited to between one and three. The electron microscope results suggest that the expression of flagella has diminished in the *mraW* deletion mutant under the culture condition used in this study.

### The Effect of *mraW* on Genome Wide DNA Methylation

**FIGURE 2:**
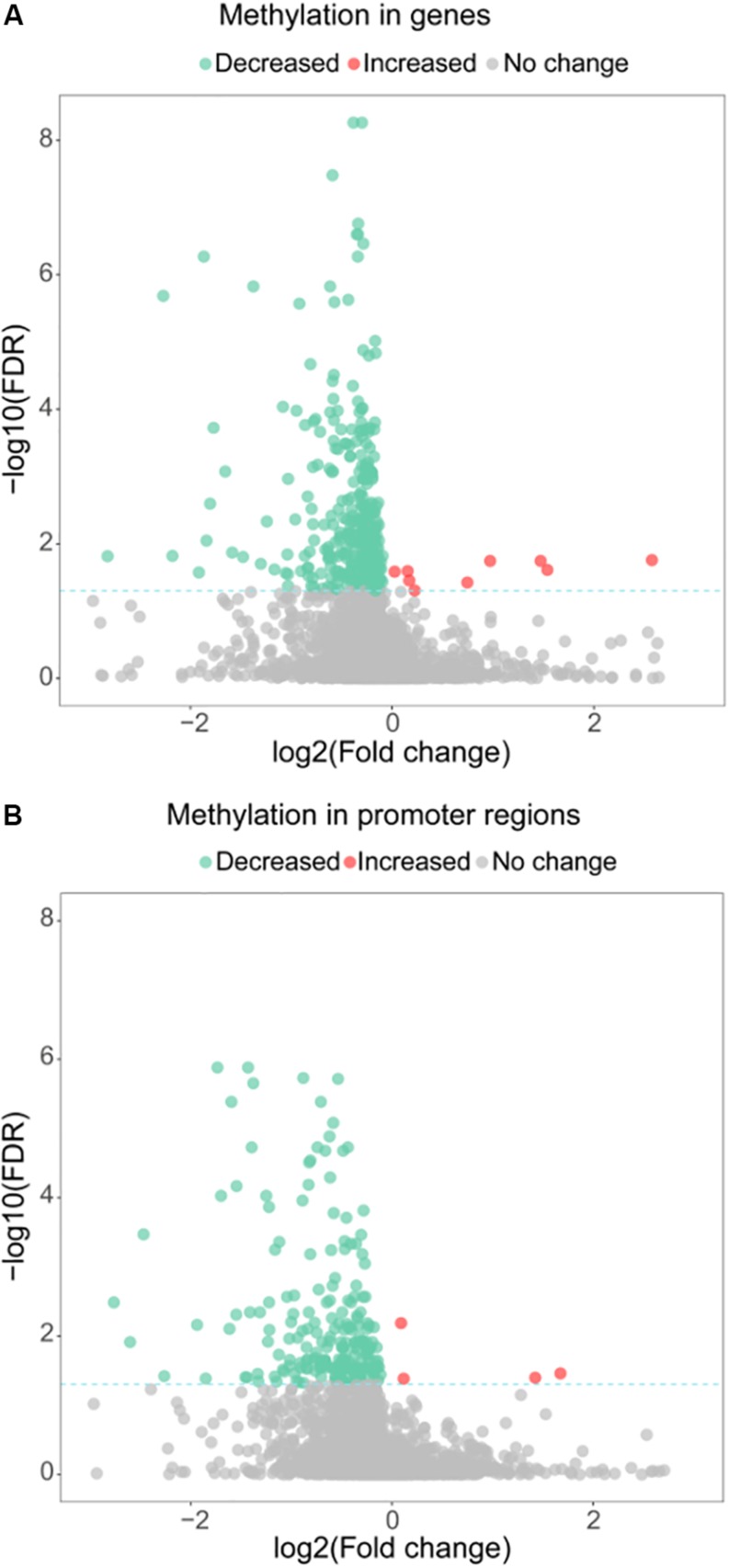
Differential methylation between EDL933 and EDL933ΔmraW. **(A)** The methylation difference between EDL933 and EDL933ΔmraW in genes. The log2 difference (EDL933ΔmraW versus EDL933) of methylation (*x*-axis) by -log10 (false discovery rate [FDR]) (*y*-axis) is shown. The broken green line shows the significance cutoff corresponding to an FDR less than 0.05. Red spots indicate genes with increased methylation; green spots indicate genes with decreased methylation. **(B)** The methylation difference between EDL933 and EDL933ΔmraW in promoter regions. The log2 difference (EDL933ΔmraW versus EDL933) of methylation (*x*-axis) by -log10 (FDR) (*y*-axis) is shown. The broken green line shows the significance cutoff corresponding to an FDR less than 0.05. Red spots indicate promoter regions with increased methylation; green spots indicate promoter regions with decreased methylation.

Besides its function as a RNA methyltransferase targeting16S RNA, we investigated whether MraW affects DNA methylation at the genome level. Genome-wide methylation profiling was performed in wild-type EDL933 and EDL933ΔmraW by bisulfite sequencing to obtain detailed information on the methylation status of each cytosine. A total of 1.2 gigabytes of sequence data were obtained for each strain. The sequencing depth of EDL933 and EDL933Δmra was 104.24- and 99.96-fold. The methylation level of C, CG, CHG, CHH (where H = A, T or C) in EDL933 and EDL933ΔmraW whole genomes was 1.56, 1.09, 3.84, and 0.44 and 1.42, 0.96, 3.50, and 0.41, respectively (see Supplementary Table S1). Although a similar methylation level was found in cytosine in EDL933ΔmraW compared to EDL933, there was a trend of differences in methylation levels in genes and promoter regions (500 bp upstream of the coding regions) which contained at least one differentially methylated regions (DMRs). The methylation levels of 219 promoters including 97 with known function and 336 genes including 152 with known function (FDR < 0.05, *p* < 0.005) (Figures 2A,B) in EDL933ΔmraW were lower compared with the wild type. These differentially methylated promoter regions and genes are discussed in detail below.

### The Effect of *mraW* on the Methylation of Flagella, Energy Metabolic Pathway and Other Virulence Genes

Consistent with decreased motility and flagellin secretion in the *mraW* mutant, two flagellar genes, *fliJ* (encoding a cytoplasmic flagellar protein) and *fliR* (flagellar apparatus integral membrane protein) and two promoters of flagellar genes, *fliK* (flagellar hook length) and *fhiA* (flagellar apparatus integral membrane protein) showed a lower level of methylation in the mutant compared to the wild type (Supplementary Tables S2, S3).

In addition, the methylation level of the promoter of *qseB*, a flagellar related quorum sensing regulator gene, was decreased in the *mraW* mutant. QseBC regulates flagella indirectly as an enhancer of flagellar master regulator FlhDC and in the absence of QseC, phosphorylation of QseB can act as a repressor of the flagellar expression (Hughes et al., 2009).

In addition to flagella related genes, the largest proportion of DMRs are related to energy metabolism pathways. 30% (29/97) promoters and 26% (39/152) genes with DMRs participate in energy metabolism accounting for the highest proportion in the whole genome DMRs (Supplementary Tables S2, S3). The methylation level of *barA* was also reduced which encodes one of the members of the BarA-UvrY two component system and plays an essential function in metabolic adaptation by controlling the carbon storage regulation system, Csr (Pernestig et al., 2003).

Many virulence genes and promoters in the *mraW* mutant were also affected at methylation level (Supplementary Tables S2, S3), including three genes of the T3SS (*escD*, *espB*, and *z4187*), three genes encoding three heat shock proteins (HtrC, Ddg, and GrpE), a cold shock protein gene (*cspA*), a helicase gene (*recD*), and a curli activator gene (*crl*). A very small number of genes/promoters (14) had increased methylation level in the *mraW* mutant (Figures 2A,B). Most of these genes were of unknown function. The five genes with known functions were *z1799*-encoding a prophage CP-933N encoded membrane protein, *tyrP*-tyrosine-specific transport system, *cspA*-transcriptional activator of HNS, *frwD*-PTS system fructose-like IIB component 2 and *eno*-enolase.

### Validation of Bisulfite Sequencing Results

To validate the genome bisulfate sequencing results, nine regions (four genes and five promoters) including *fliR*, *fliJ*, *z2975*, *P*_*fliK*_, *P*_*fhiA*_, *P*_*yidP*_, *P_*treR*_, z1440*, and *z4981* were selected for bisulfite sequencing PCR (BSP). Among these DMRs, *z1440*, and *z4981* showed increased methylation level. The methylation levels were higher by BSP in both the wild type and the *mraW* mutant, but the magnitude of reduction of methylation (38% reduction on average) in the mutant is similar to genome bisulfate sequencing results (30% reduction on average). These BSP data analyses revealed highly similar methylation patterns compared with genome bisulfate sequencing results including DMRs with increased methylation level (Supplementary Table S4).

### Motifs of DMRs and MraW Binding to DMRs of Flagellar Gene Promoters and Coding Sequences

**FIGURE 3:**
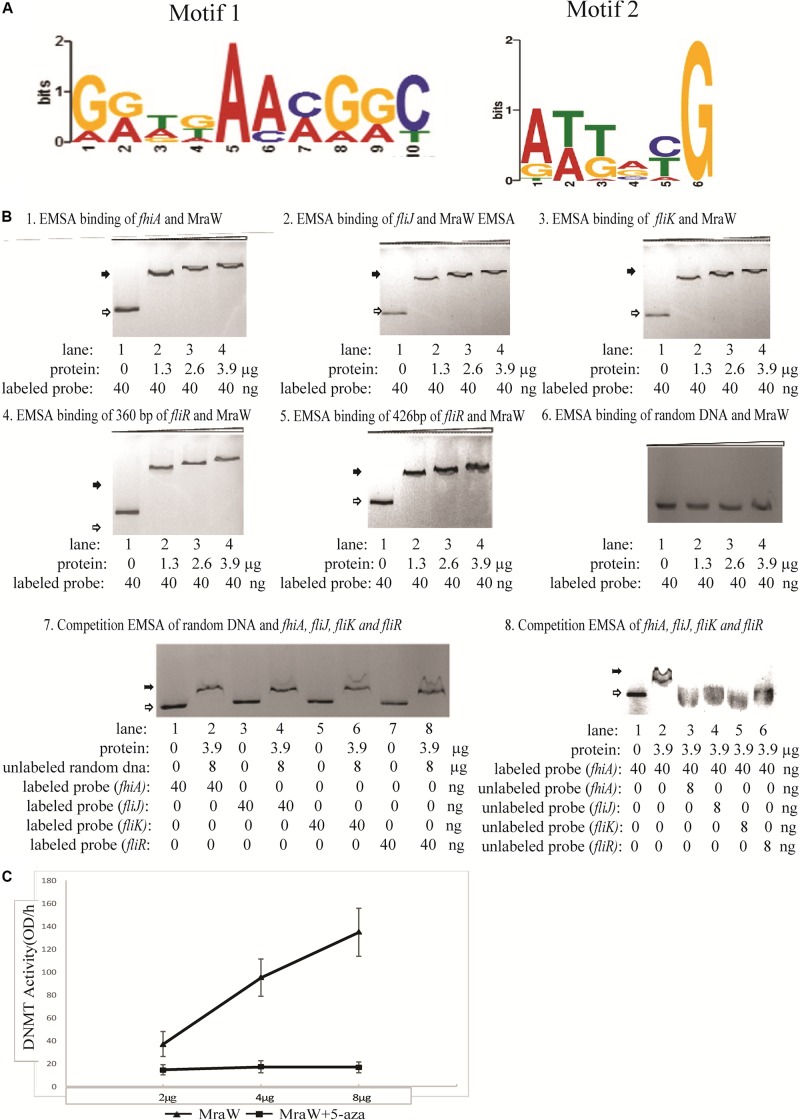
The methylation effect of MraW and binding interaction with flagellar genes or promoter regions. **(A)** DNA binding motif of *mraW*. DNA binding motif of mraW in flagellar gene related DMRs was analyzed using MEME program and a common flexible motif from six DMRs was found: GGTGAACGGC (left). DNA binding motif of *mraW* in heat shock protein gene DMRs was analyzed using MEME program and a common motif from 21 DMRs was found: ATTACG (right). **(B)** Assessment of MraW binding to the DMRs of flagellar gene and promoter fragments and random DNA sequence without potential motifs. Reaction constituents are indicated underneath. Open arrow indicates free labeled DNA, the black arrow indicates MraW-DNA complexes. **(1–5)** EMSA binding assay between *fhiA*, *fliJ*, *fliK* and *fliR*, and MraW. **(6)** EMSA binding assay between random DNA and MraW. **(7)** Competition EMSA was carried out between random DNA sequence and Z0290 (*fhiA*), Z3032 (*fliJ*), Z3033(*fliK*), or Z3040 (*fliR*). Labeled Z0290 (*fhiA*), Z3032 (*fliJ*), Z3033(*fliK*), and Z3040 (*fliR*) was incubated with the presence of 200-fold molar excess of unlabeled random DNA sequence. **(8)** The competition assays were also carried out between MraW and flagellar sequences to test the binding intensity. Labeled Z0290 (*fhiA*) probe was incubated with presence or absence of 200-fold molar excess of unlabeled probe Z0290 (*fhiA*), Z3032 (*fliJ*), Z3033(*fliK*), or Z3040 (*fliR*). **(C)** MraW DNA methylation activity. DNMT activity increased with higher concentration of MraW proteins and repressed by DNA methylation inhibitor 5-aza.

Given the methylation effect is genome wide, we searched for common motifs in the DMRs of the whole genome. No common motif with a *p-*value < 0.0001 was found. We then restricted the searches to the DMRs of the flagellar genes and heat shock protein genes. In the four flagella genes, three genes *fliJ*, *fliK*, *fhiA* containing six DMRs while *fliR* had no DMR. One motif, GATGAAAGGC, was common in these six DMRs with a *p-*value < 0.0001 which was flexible as shown in Figure 3A. Similarly, common motifs in 21 DMRs of the three heat shock protein genes *htrC*, *ddg*, and *grpE* were investigated. One common motif, ATTACG, was found with *p-*value < 0.0001 (Figure 3A). Since ATTACG occurs 1330 times in the EDL933 genome, we did not pursue this motif further.

The DMRs of flagellar sequences and potential common motifs clearly indicated the possibility of physical interaction between MraW and flagellar sequences. Therefore, MraW was expressed and purified for electrophoretic mobility shift assay (EMSA) to assess its binding ability. The prepared protein was identified by high-performance liquid chromatography-electro-spray ionization-mass spectrometry (HPLC-ESI-MS) after trypsin digestion. The protein score was 795.72 comparing with standard protein database (data not shown). The relative purity was 93.4% (peak area ratio) determined by high pressure liquid chromatography with UV Detector (HPLC-UV) method (Supplementary Figure S1). Four flagellar nucleotide sequences namely, promoters of *fhiA* and *fliK*, and coding sequences of *fliJ* and *fliR* including the motif were cloned and used for the assay (Supplementary Table S5). Three protein concentrations were tested. Binding occurred at the lowest concentration with major shift of mobility. Increasing concentrations of MraW led to small decreases in mobility (Figures 3B1–5). The binding results suggest that MraW could directly affect the expression of these flagellar genes. A random DNA sequence without potential motifs was used as negative control. EMSA results showed low binding of MraW to the random sequence indicating that the binding of MraW to flagellar gene sequences were sequence/motif specific (Figure 3B6). Competition EMSA assay was performed between random sequence and the other four flagellar sequences showing obvious shift of labeled flagellar with presence of unlabeled random DNA which confirmed the specific binding between MraW and the four flagellar sequences (Figure 3B7). The observed shift in the reaction of MraW and flagellar sequences with presence of 200-fold molar excess random DNA indicated no or low binding of MraW and random control DNA. Competition EMSA assay was also carried out to test the intensity among flagellar sequences. The signal intensity of shifted bands for Z02090 (*fhiA*) was reduced upon addition of unlabeled probe of Z0290 (*fhiA*), Z3032 (*fliJ*), Z3033(*fliK*), and Z3040 (*fliR*) as expected. Competition EMSA assay results showed low/no binding of MraW to the labeled probe by adding unlabeled *fhiA* and *fliJ*, *fliK* probe indicating that the binding of MraW to *fliJ*, *fliK* sequences was equal to *fhiA* (Figure 3B8).

### DNMT Activity of MraW

Since MraW can directly bind to the flagellar gene sequences, we tested whether MraW methylates DNA. A commercial ELISA kit containing DNMT substrate (cytosine) and anti-5-methylcytosine antibody was used for DNMT activity measurement. The purity of the MraW we produced was 93.4% (Supplementary Figure S1). MraW protein with different concentrations with or without DNA methylation inhibitor 5-aza was added and incubated with the DNMT substrate. Methylated DNA can be recognized with the anti-5-methylcytosine antibody which was attached with an enzyme catalyzing the substrate to blue color. DNMT activity of MraW ranged from 37 OD/h to 137 OD/h when the MraW protein concentration increased from 2 μg to 8 μg (Figure 3C). The DNMT activity of MraW was repressed by 5-aza (Figure 3C). In addition, we tested the effect of DNA methylation inhibition *in vivo* by adding 5-aza to the culture of *mraW* complement strain (pBADmraW+5-aza) and measuring motility difference with and without 5-aza. The motility was repressed with the addition of 5-aza (Figure 1A). Hence we conclude that there is a positive correlation between the DNMT activity and MraW protein concentration indicating a DNA methylation function of MraW. It should be noted that it is unlikely but cannot be ruled out that the 6.6% impurity of the MraW preparation may contribute to the enzymatic activity measured.

### Influence of *mraW* on Colonization in Mice

**FIGURE 4:**
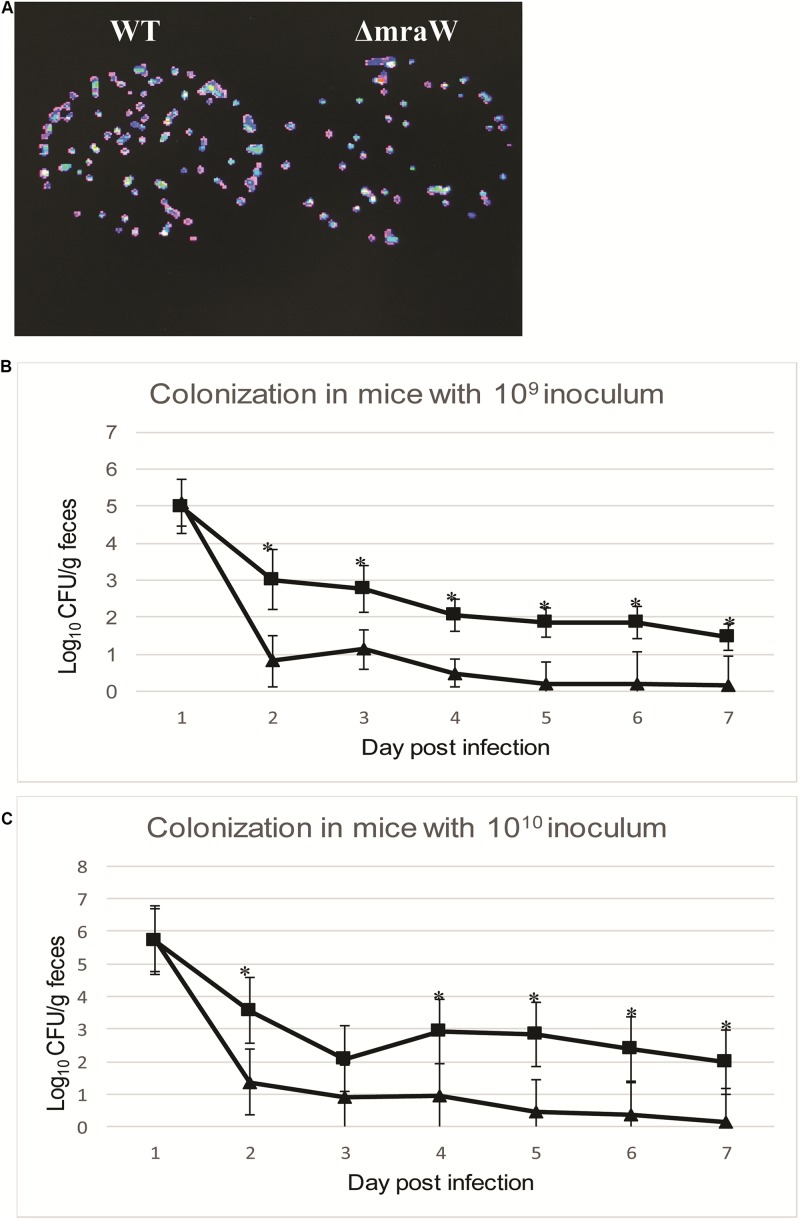
Colonization of BALB/c mice by EDL933 and EDL933ΔmraW with luminescent plasmid pGEN-luxCDABE. **(A)** Results showed that each colony contained luminescent plasmid pGEN-luxCDABE. **(B)** Colonization levels of EDL933 and EDL933ΔmraW over 7 days after oral gavage with a 10^9^ CFU mixed inoculum (dashed lines with ■ for EDL933 and ▲ for the *mraW* mutant. **(C)** Colonization levels of EDL933 and EDL933ΔmraW over 7 days after oral gavage with a 10^10^ CFU mixed inoculum (dashed lines with ■ for EDL933 and ▲ for the *mraW* mutant).

We next asked whether *mraW* would affect the colonization of EDL933 *in vivo* using the mouse model. To assess the virulence effect *in vivo*, a constitutively luminescent plasmid was introduced into the wild type and the mutant to identify them in the mixed infection experiments. Six-week-old female BALB/c mice were used. Two groups of 10 mice were intragastrically administered approximately 10^9^ and 10^10^ CFU of equal mix of wild type EDL933 and the *mraW* deletion mutant. We used two different inoculum sizes as better colonization was observed in high inoculum (above 10^9^ CFU) in previous studies (Mohawk et al., 2010; Zumbrun et al., 2013). The maintenance of the bioluminescent plasmid was assessed by luciferase scanning. The degree of bacterial colonization was measured by the number of luminescent bacteria present per gram of feces. Mice was fed the same numbers of bacteria of the EDL933 and EDL933ΔmraW. Fecal shedding was examined up to day 7. Each colony from the shedding fecal sample was luminescent on Sorbitol–MacConkey agar plates with ampicillin for selection of *E. coli* O157:H7 (Figure 4A). The *mraW* deletion mutant was selected by kanamycin resistance. A very similar shedding level of these two strains were found at day 1. A significant difference in colonization between EDL933 and EDL933ΔmraW was found from day 2 to day 7 (Figures 4B,C). A 100 times difference was found between the wild type and the *mraW* deletion mutant from day 2 in both 10^9^ and 10^10^ CFU inoculum groups. The results indicated that the *mraW* deletion mutant colonized the mice poorer than the wild type EDL933.

### The Effect of *mraW* on the Expression of a Selected Set of Genes

**FIGURE 5:**
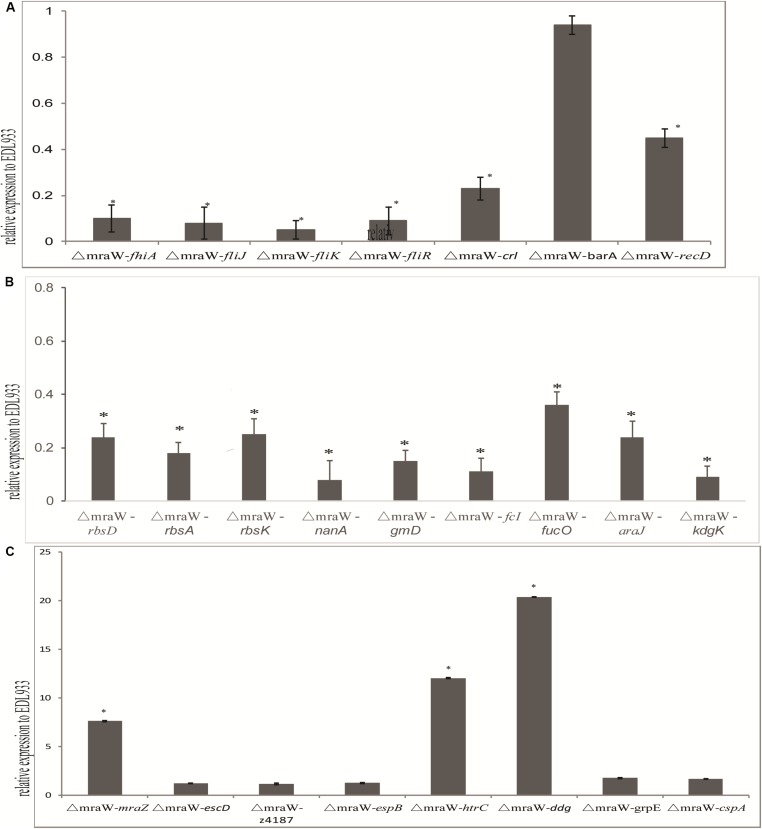
Effect of *mraW* on gene expression at transcriptional level and on T3S profile. Relative mRNA expression of selected genes was normalized to that of the housekeeping gene *gapA*. Results represent mean values ± standard deviations (SD) for three independent experiments. Differences were analyzed for significance using *t*-test with significant difference between two strains (*P* < 0.01). **(A)** Motility genes including *fhiA*, *fliJ*, *fliK*, *fliR*, *crl*, *barA*, and *recD* that were downregulated in mRNA expression compared to wild type strain EDL933. **(B)** Metabolic genes including *rbsD, rbsA*, *rbsK*, *nanA*, *gmD*, *fcI*, *agaI-2*, *galP*, *fucO*, *araJ*, and *kdgK* that were upregulated in mRNA expression compared to wild type strain EDL933. The expression of the gene in EDL933 was set to one. **(C)** Genes including *mraZ*, *escD*, *z4187*, *espB*, *htrC*, *ddg*, *grpE*, and *cspA* that were upregulated in mRNA expression compared to wild type strain EDL933. The expression of the gene in EDL933 was set to one.

We investigated the effect of *mraW* deletion on a selected set of genes which are related to virulence phenotype, primarily based on methylation effect detected above. The mRNA expression of flagella related genes *fliJ*, *fliR*, *fliK*, and *fhiA* were all decreased (*t*-test, *P* < 0.01) (Figure 5A), which is consistent with the decline of flagellin protein production/secretion. Curli related gene, *crl* and the helicase gene, *recD*, were also decreased at the transcriptional level (*t*-test, *P* < 0.01) (Figure 5A). However, three genes encoding heat shock proteins, *htrC*, *ddg*, and *grpE*, were significantly increased at the transcriptional level (*t*-test, *P* < 0.01) (Figure 5B). The mRNA transcription of cold shock protein CspA was also increased in a low but significant level (*t*-test, *P* < 0.01) (Figure 5C).

Nine genes of carbon nutrition metabolism relevant to colonization were examined with qRT-PCR (Chang et al., 2004; Leatham et al., 2005). As expected, *rbsD*, *rbsA*, *rbsK*, *nanA*, *gmD*, *fcI*, *agaI-2*, *galP*, *fucO*, *araJ*, and *kdgK* all showed a reduced mRNA expression (*t*-test, *P* < 0.01) (Figure 5C).

Type III secretion phenotype was investigated in the mutant. The transcription levels of *escD*, *espB*, and *z4187* were not increased significantly (Figure 5C). Consistently, no difference was found between the wild type and the mutant for EspB at protein level for which we performed western blotting analysis (data now shown).

Due to the antagonistic functions of *mraW* and *mraZ* and their effect on virulence, the expression of *mraZ* was also examined. *mraZ* expression showed more than seven times increase in the *mraW* mutant compared to the wild type (Figure 5C), confirming the antagonistic effect previously observed (Eraso et al., 2014).

## Discussion

In this study, we investigated the role of MraW in genome-wide DNA methylation and its role in virulence in *E. coli* O157:H7. We found the *mraW* deletion mutant has a genome wide effect on methylation levels of a large number of genes including flagellar related genes, metabolic and respiratory pathway genes, curli related genes, T3SS genes and other virulence related genes. We also found that reduced methylation was correlated with altered expression of 21 of the 24 genes tested. MraW was found to have a profound effect on the transcription and protein expression of flagellar genes and motility. MraW was also found to be able to bind to DMRs of flagellar genes and their promoters and methylate cytosine *in vitro*.

The rRNA MTases have been mainly involved in ribosome biogenesis and translation fidelity (Das et al., 2008; Burakovsky et al., 2012). Some rRNA MTases have been reported to play a role in antibiotic resistance and stress response (Helser et al., 1971; Gustafsson and Persson, 1998; Bujnicki and Rychlewski, 2001; Andersen and Douthwaite, 2006; Lesnyak et al., 2006; Toh et al., 2008; Nichols et al., 2011; Osterman et al., 2011; Basturea et al., 2012). The 16S rRNAMTase encoded by *mraW* is known to be involved in cell division and PG synthesis (Kimura and Suzuki, 2010). Recently, *mraW* (*rsmH*) was shown to increase the virulence of *S. aureus in vivo* (Kyuma et al., 2015). It was found that the methylation of C1412 of the 16S rRNA which corresponds to C1402 of 16S rRNA in *E. coli* enhanced the animal lethality of *S. aureus* due to increased resistance to oxidative stress in host animals (Kyuma et al., 2015). These observations suggest that *mraW* has a wider role than 16S rRNA methylation and cell division.

MraW participated in DNA methylation at whole genome level. A total of 219 promoters and 336 genes showed a decreased methylation level in △*mraW*. The DNMT activity of MraW was detected *in vitro* using a cytosine methylation assay and repressed by the DNA methylation inhibitor a-5za, confirming its DNA methyltransferase function. A linear correlation between DNMT activity and protein concentration was found. In addition, we found that MraW can directly bind DNA sequences with DMRs, suggesting that MraW can directly bind to DNA. The binding between MraW and flagellar sequences was specific as indicated by random control DNA sequence with presence or absence of labeled flagellar sequence. In addition, the binding intensity between MraW and three flagellar sequences (*fhiA* and *fliJ*, *fliK*) are very similar as demonstrated by competition EMSA assay. The default substrate of MraW is the 30S subunit when it functions as an RNA MTase. The crystal structure of MraW shows two distinct but structurally related domains: the typical MTase domain and the putative substrate recognition and binding domain (Wei et al., 2012). The purification of MraW was tested by HPLC-ESI-MS and HPLC-UV. The protein score and the relative purity results suggested that our assay was largely measuring MraW activity rather than the activity of contaminated proteins. It is unlikely but cannot be ruled out that the 6.6% impurity contributing to the enzymatic activity measured. It remains to be investigated whether the substrate recognition and binding domain recognizes DNA. The MraW crystal structure contains a cytidine binding site (Wei et al., 2012). Eight hydrophobic residues including Trp139, Ala143, Ala146, Tyr155, Trp211, Val212, Ile148, and Leu152 interacts indirectly with cytidine forming a hydrophobic environment via a water molecule. Therefore, there is a possibility that the cytosine from DNA sequences could be recognized and methylated by MraW.

Our results showed that MraW affected flagellar motility directly through regulation of the expression of flagellar genes. A potential binding motif and direct binding were found between MraW protein and flagellar related nucleotide sequences including *fliJ*, *fliR*, *fhiA*, and *fliK*, indicating a direct physical interaction between MraW and these DNA sequences. The flagellum is composed of three parts: the basal body, the hook, and the filament (Macnab, 2003; Barker et al., 2014). FliR is one of the six integral membrane proteins of the export apparatus. FliJ participates in forming an ATPase ring complex at the export gate that also plays an important role in substrate recognition with FliI (Minamino, 2014; Sajo et al., 2014). FliK takes part in switching flagellar secretion mode from hook construction to filament elongation and controlling the length of the hook (Uchida and Aizawa, 2014). The diminished flagella on the cell surface in the *mraW* mutant is possibly related to the repression of *fliK*. *fhiA* is a homolog of *flhA* which coordinates the export of flagellins (Bange et al., 2010; Ibuki et al., 2013). The methylation level of *qseB* was also decreased in the mutant. However, QseB represses the motility only in the absence of QseC. Taken together, *mraW* exerts its effect on flagellin production/secretion and motility by acting on the basal apparatus, hook construction, filament length and secreted proteins.

From the mouse colonization results, we found a decreased level of colonization by the *mraW* mutant using BALB/c mice, a model firstly developed by Mohawk et al. (2010) and Mohawk and O′Brien (2011). A lower colonization by the *mraW* mutant is most likely due to nutritional constraints. Carbon nutrition metabolism was found to have essential effect in the mouse colonization of *E. coli* (Peekhaus and Conway, 1998; Chang et al., 2004) and sialic acid, ribose, mannose and fucose are involved in the colonization of *E. coli* in mice (Chang et al., 2004; Leatham et al., 2005). We found many genes that were decreased both in methylation and transcriptional levels in the *mraW* mutant are related to energy metabolism mainly related to carbon nutrition metabolism including *rbsD*, *rbsA*, and *rbsK* for ribose metabolism, *nanA* for sialic acid, *gmD* for mannose, *fcI* and *fucO* for fucose. Other carbon metabolism genes include *agaI-2* for isomerase, *araJ* for arabinose, and *kdgK* for hexuronates. In addition, the methylation and transcription levels of *crl* were reduced in the *mraW* mutant compared to the wild type. As an activator of curli production, Crl binds to stationary phase sigma subunit of RNA polymerase directly (Bougdour et al., 2004). Hence a reduced expression of *crl* might affect colonization as well via curli which can assist the colonization of *E. coli* (Lloyd et al., 2012). The decreased colonization by the *mraW* mutant is less likely due to reduction of motility as less or non-motile *E. coli* O157:H7 was preferred in the animal intestine including mice (Leatham et al., 2005; Sharma and Casey, 2014).

Overall, we found a positive correlation between alteration of methylation levels in genes or promotors and levels of transcription except T3SS genes in the *mraW* mutant, suggesting that *mraW* plays a role in genome wide DNA methylation either directly or indirectly and consequently affects transcription and function of a large number of genes. There are a number of possible mechanisms. Firstly, MraW may have a dual function as a DNA methylase that directly methylate DNA in regions where it can bind. A direct binding of MraW protein to flagellar coding or promoter regions was found indicating a physical interaction between MraW and DNA sequences. Common motifs were also found in a subset of genes such as a motif in the six DMRs from three genes *fliJ*, *fliK*, and *fhiA*. However, no common motifs were found across the large number of genes that have been affected. Secondly MraW may have affected the expression of other methylases that in turn affected the methylation of other genes. Thirdly, the effect of MraW might be achieved via MraZ. The deletion of *mraW* perturbs *mraZ* which is antagonistic to *mraW* and a known regulator of *mraW* and 100 other genes (61 activated and 31 repressed) in *E. coli* K-12 (Eraso et al., 2014). However, when *mraZ* was mildly over produced it affected the expression of 970 genes in *E. coli* K-12 (Eraso et al., 2014). Hence, *mraW* might control genome wide gene expression via the antagonistic relationship with *mraZ*. We found the expression of *mraZ* was increased in the *mraW* mutant which is consistent with the observations in *E. coli* K-12 (Eraso et al., 2014). Fourthly, the RNA methyltransferase function of MraW can affect the expression of a large range of genes (Kyuma et al., 2015). We cannot rule out this effect as no RNA methyltransferase inhibitor was available.

Although the vast majority of the differentially methylated genes/promoters had reduced methylation in the *mraW* mutant, 13 showed increased methylation levels. A plausible explanation for these small number of genes with increased methylation is that these DMRs may be methylated by other methylases that were repressed by *mraW*. It is unlikely all of these were false positives as two were confirmed by BSP PCR sequencing. Further studies will be required to elucidate the mechanisms involved.

In conclusion, we found that *mraW* plays a role in gene regulation through DNA methylation in addition to its known function in methylating 16S rRNA to increase mRNA decoding fidelity. *mraW* affected DNA methylation, motility, and mouse colonization and clearly plays a role in virulence in *E. coli*.

## Materials and Methods

### Bacterial Strains, Plasmids and Bacterial Culture

Details of bacterial strains and plasmids used in this study are listed in Supplementary Table S5. Bacteria were routinely cultured in Luria-Bertani (LB, Miller) broth or agar. DNA methylation inhibitor, 5-aza, was purchased from Sigma-Aldrich. Antibiotics were included when required at the following concentrations: 100 μg ml^–1^ ampicillin, 50 μg ml^–1^ kanamycin, and 50 μg ml^–1^ chloramphenicol. Other chemicals added to media were 0.2% L-arabinose, bromo-chloro-indolyl-galactopyranoside (X-gal) 20 μg ml^–1^ (dissolved in dimethylformamide) and Isopropyl β-D-1-thiogalactopyranoside (IPTG) 2 μg ml^–1^. Most of motility related experiments were performed at an OD of 0.6 at which density bacterial growth is in the log phase.

### Construction of *mraW* Deletion Mutant in EDL933

Construction of the *mraW* deletion mutant in EDL933 was performed using one step method as described by Datsenko and Wanner (2000). The *kmr* gene was amplified by PCR from plasmid pRS551 (Simons et al., 1987) with primer pair P1 and P2 (Supplementary Table S6) (Oka et al., 1981). EDL933ΔmraW mutant was confirmed by PCR and sequencing. The primer pairs P3 and P4, P5 and P6 (Supplementary Table S6) were used to confirm *mraW* gene deletion.

### Construction of Plasmids for Expression and Complementation

Complementation plasmid pBADmraW was constructed using PCR product amplified with high fidelity Phusion polymerase cloned into pBAD/Myc-HisA (Supplementary Table S5). Primers including 5-mraWF and 3-mraWR were used (Supplementary Table S6). *E. coli* strain DH5a was used as the intermediate host strain for cloning and all constructs were verified by sequencing.

### Motility Assays

Swimming motility was evaluated as described by Xu et al. (2013). Briefly, wild type, the *mraW* mutant, the complement strain and complement strain with 5-aza (10 μM) on plates were cultured overnight and stab inoculated with a sterile inoculating needle and incubated at 37°C for 16 h. All strains were tested in triplicate and each experiment was carried out on three separate occasions. The motility radius of each strain was measured and analyzed by *t*-test.

### Transmission Electron Microscopy

Transmission Electron Microscopy was carried out as previously described (Xu et al., 2013). In brief, EDL933 and its mutant EDL933ΔmraW were cultured in LB broth with shaking till optical density (OD_600_) of 1.0. Bacteria on TEM grids were stained with 1% (wt/vol) phosphotungstic acid and examined with a Philips Tecnai 12 transmission electron microscope. Images were obtained with Gatan Digital Micrograph Imaging System by an Erlang Shen CCD camera (Gantan).

### Preparation of H7 Supernatant Protein

To examine the H7 Flic protein expression in the wild type and the mutant strains, bacteria were cultured overnight in LB at 37°C with shaking and sub-cultured in a dilution of 1:100 in LB. Strains were grown to a final OD_600_ of 1.0 and then centrifuged at 4000 *g* for 30 min at 4°C and supernatants were filtered through 0.45 mm low protein-binding filters (Millipore). A 10% (v/v) final concentration of trichloroacetic acid (TCA; Sigma-Aldrich) was used to precipitate the proteins. Supernatants were incubated overnight at 4°C for thorough precipitation and centrifuged at 4000 *g* for 30 min at 4°C. Protein pellets were air-dried and resuspended in an appropriate volume of resuspension buffer (1.5 M Tris–HCL) in order to standardize samples and to take into account the slight variation in OD_600_ at which cultures were harvested.

### H7 Flic Protein Detection

Supernatant protein was resolved on a 12% sodium dodecyl sulfate-polyacrylamide (SDS-PAGE) gel. SDS-PAGE separated proteins were transferred onto Hybond ECL nitrocellulose membrane (Amersham Biosciences) with a *Trans-*Blot electrophoretic transfer cell (Bio-Rad). Nitrocellulose membranes were blocked with 8% (w/v) dried milk powder (Marvel) in PBS at 4°C overnight and incubated with the relevant antibodies diluted in wash buffer 0.05% (v/v) polyoxyethylene sorbitan monolaurate (Tween 20, Sigma-Aldrich) in PBS at the following dilutions: primary antibodies against H7 flagellin (Statens Serum Institut, Denmark) were diluted 1:1000 and secondary antibody with IR Dye 800-labeled anti-rabbit IgG (Rockland, Gilbertsville, PA, United States) were diluted 1:10000. Fluorescence signal was captured by Odyssey imager (LI-COR).

### DNA Sequencing, Library Preparation and Genome Methylation Sequencing

Genomic DNA was extracted with Wizard Genomic^®^ DNA Purification Kit (Promega). DNA was sonicated to 100–300 bp fragments. Bisulfate treatment was carried out by ZYMO EZ DNA Methylation-Gold kit (Zymo Research). By gel purification, DNA fragments with proper size were used for PCR amplification. The proper size PCR-amplified fragments were sequenced using HiSeq2000. Sequencing data was mapped onto the reference *E. coli* O157:H7 strain EDL933 to obtain the genome methylation data.

### Validation of Genome Bisulfite Sequencing Results by Bisulfite Sequencing PCR

Genomic DNA bisulfate treatment was carried out by EpiTect^®^ Bisulfite kit (Qiagen). Primers for Bisulfite sequencing PCR were designed using Methyl Primer Express v1.0 (Supplementary Table S6, from fhiAP1F to treR2R). PCR was carried out using the DNA template which was treated by EpiTect^®^ Bisulfite kit. PCR product was cloned into pMD-18-T and sequenced. For each DNA fragment, at least 10 clones were selected for sequencing to minimize the sequencing error. Cytosine methylation data was achieved by mapping the sequencing data to the reference *E. coli* O157:H7 str. EDL933.

### RNA Extraction, cDNA Synthesis and Quantitative Reverse Transcription PCR

Overnight cultures of *E. coli* EDL933 and, its mutant strain was diluted 100-fold in LB broth and then grown to an OD_600_ of 0.6 with shaking. Total RNA was extracted using RNeasy Mini Kit (Qiagen) following the manufacturer’s instructions. RNA was treated with DNase I (NEB). Expression of *fhiA*, *fliJ*, *fliK*, *fliR*, *crl*, *barA*, *recD*, *mraZ*, *escD*, *z4187*, *espB*, *htrC*, *ddg*, *gprE*, *cspA rbsD, rbsA*, *rbsK*, *nanA, gmD, fcI*, *agaI-2*, *galP*, *fucO*, *araJ*, and *kdgK* were quantified by quantitative reverse transcription-PCR (qRT-PCR) analysis. Reverse transcription was performed using PrimeScript^®^ RT reagent Kit (Perfect Real Time) (TaKaRa). qRT-PCR was carried out using SYBR^®^ Premix Ex TaqTM II (Perfect Real Time) (TaKaRa) using a Rotor-Gene Q thermal cycler (QIAGEN). Data was analyzed with Rotor-Gene Q Series Software, version 1.7 (QIAGEN). Data were normalized to the endogenous reference gene *gapA* and analyzed by the cycle threshold method (2-△△CT) (Livak and Schmittgen, 2001). Three independently isolated cDNA samples were analyzed. Primers for amplifying *gapA*, *fhiA*, *fliJ*, *fliK*, *fliR*,*crl*, *barA*, *recD*, *mraZ*, *escD*, *z4187*,*espB*, *htrC*, *ddg*, *gprE*, *cspA rbsD, rbsA*, *rbsK*, *nanA, gmD, fcI*, *agaI-2*, *galP*, *fucO*, *araJ*, and *kdgK* are detailed in Supplementary Table S6 (From fhiA RT F to kdgK RT R).

### EMSA Test

The promoter of *fhiA* and *fliK*, genes of *fliJ* and *fliR* and random DNA sequence (Supplementary Table S6) were synthesized and cloned into pUC19-c vector with DH10b competent cell. As the sequence of the *fliR* gene exceeds 500 bp, it was divided two frarments. The cloned sequences and constructed plasmids were listed in Supplementary Table S5. Plasmid pDS17039, pDS17040, pDS17041, pDS17042, pDS17043, and pDS17045 were achieved, respectively, from the sequence of *fhiA*, *fliK*, *fliJ*, *fliR* and random DNA sequence. For preparation of fluorescent FAM labeled probes, the promoter region of pDS17039 plasmid, pDS17040 plasmid, pDS17041 plasmid, pDS17042 plasmid, pDS17043 plasmid and pDS17045 plasmid, was PCR amplified with Dpx DNA polymerase using primers of M13F (FAM) and M13R. The FAM-labeled probes were purified by the Wizard^®^ SV Gel and PCR Clean-Up System (Promega, United States) and were quantified with NanoDrop 2000C (Thermo Fisher Scientific, United States). MraW protein were prepared and purified as previously described (Wei et al., 2012). EMSA was performed in a 20 μl reaction volume that contains 40 ng probe and varied of MR proteins, in a reaction buffer of 50 mM Tris–HCl [pH 8.0], 100 mM KCl, 2.5 mM MgCl2, 0.2 mM DTT, 2 μg salmon sperm DNA and 10% glycerol. After incubation for 20 min at 25°C, the reaction system was loaded into 2% TBE gel buffered with 0.5 × TBE. Gels were scanned with Image Quant LAS 4000 mini (GE Healthcare).

Competition EMSA was carried out between random DNA sequence and Z0290 (*fhiA*), Z3032 (*fliJ*), Z3033(*fliK*) and Z3040 (*fliR*). Labeled Z0290 (*fhiA*), Z3032 (*fliJ*), Z3033(*fliK*) or Z3040 (*fliR*) was incubated with the presence of 200-fold molar excess of unlabeled random DNA sequence. The reaction mixture was resolved with gels as described above. The binding intensity of MraW and flagellar sequences binding was also examined by competition assays. Labeled Z0290 (*fhiA*) probe was incubated with presence or absence of 200-fold molar excess of unlabeled probe Z0290 (*fhiA*), Z3032 (*fliJ*), Z3033(*fliK*) or Z3040 (*fliR*). The reaction mixture was resolved with gels as described above.

### DNMT Activity Test

MraW protein were prepared and purified as previously described (Wei et al., 2012). DNMT activity test was performed in accordance with EpiQuik^TM^ DNMT Activity/Inhibition Assay Ultra Kit manual (Colorimetric) (Epigentek). DNMT activity was calculated as the following formula:

$$D\,N\,M\,T\,A\,c\,t\,i\,v\,i\,t\,y=\frac{\left(S\,a\,m\,p\,l\,e\,O\,D-B\,l\,a\,n\,k\,O\,D\right)}{\left(h\,o\,u\,r^{*}\right)}\times 1000$$

^∗^Incubation time of protein and DNMT substrate (cytosine).

The DNA methylation inhibitor 5-aza was used in a final concentration of 10 μM as required.

### Identification of MraW Key Motifs

To examine the potential interacting motif of *mraW* in whole genome, flagellar and heat shock genes, the motifs in all DMRs were searched by meme 4.8.1 software. Motifs with *p-*value < 0.0001 were selected.

### Ethics Statement

All animal work was approved by Laboratory Animal Welfare and Ethics Committee at Chinese National Institute for Communicable Disease Control and Prevention. The project license number assigned by the Laboratory Animal Welfare and Ethics Committee is 20171030. Animal experiments were performed in accordance with the protocol approved by the Laboratory Animal Welfare and Ethics Committee in IVDC, China CDC.

### Mouse Infection Studies

To construct selective bacteria maintaining *in vivo* in the absence of antibiotic pressure, a constitutive plasmid pGEN-luxCDABE encoding bacterial luciferase was electro-transformed into the wild type and the *mraW* deletion mutant (Lane et al., 2007). Conventional mice model was successfully used for intestinal colonization with high doses of *E*. *coli* O157:H7 (Mohawk and O′Brien, 2011). Six-week-old female BALB/c mice from Charles River (China, Beijing) was infected by gavage with 10^9^ and 10^10^ CFU of the wild type and the mutant both containing pGEN-luxCDABE. To prepare the inoculum, overnight culture at 37°C with shaking at 225 rpm in LB media with 100 μg/ml ampicillin were pelleted by centrifugation for 10 min at 1,000 × *g*. The bacteria were then resuspended in 1/40th volume of PBS. Groups of 10 mice each were gavaged with one hundred microliters of the organisms. The day of infection was designated as day 0. The shedding extent of individual mice was monitored as below. Fecal pellets from each mouse were collected, weighed, and suspended 1:10 (wt/vol) in PBS. The fecal pellet and PBS mixtures were mixed by vertexing at room temperature for 1 min, every 10 min, for 30 min. Ten-fold dilutions of the mixtures were made with PBS, and two aliquots each containing one hundred microliters of the homogenates were plated onto Sorbitol–MacConkey agar plates containing 100 μg ml^–1^ ampicillin and 50 μg ml^–1^ kanamycin, respectively. Plates were incubated overnight at 37°C. Colonies with luminescence were imaged by using *in vivo* FX Pro (Bruker) and counted the next day.

## Data Availability Statement

All datasets generated for this study are included in the article/Supplementary Material.

## Ethics Statement

All animal work was approved by the Laboratory Animal Welfare and Ethics Committee at Chinese National Institute for Communicable Disease Control and Prevention. The project license number assigned by the Laboratory Animal Welfare and Ethics Committee is 20171030. Animal experiments were performed in accordance with the protocol approved by the Laboratory Animal Welfare and Ethics Committee in IVDC, China CDC.

## Author Contributions

XX, RL, and JX planned the experiments. XX, CW, PG, ZT, XLL, XP, HeZ, YH, YZ, XCL, and SL performed the experiments. XX, XW, DW, JP, and HoZ analyzed the data. CY and YD contributed the reagents. XX and RL wrote the manuscript.

## Conflict of Interest

The authors declare that the research was conducted in the absence of any commercial or financial relationships that could be construed as a potential conflict of interest.

## Supplementary Material

FIGURE S1 **(A)** MraW protein was purified and filtered by molecular sieve. MraW was first purified using gel filtration chromatogram (superdex 75). The single peak (78–90 ml) were collected and concentrated to 4 mg/ml. **(B)** The five tubes collected through molecular sieve were run on SDS-PAGE gel (27–31#). 27–31# is standing for the tube numbers from molecular sieve. MraW was indicated by black arrow. **(C)** The relative purity was 93.4% (peak area ratio) as determined by the HPLC-UV method.

TABLE S1 Whole genome methylation level of C, CG, CHG, and CHH. H = A, T or C.

TABLE S2 Promoter regions with DMRs between EDL and the ΔmraW.

TABLE S3 Genes with DMRs between EDL and the ΔmraW.

TABLE S4 Validation of bisulfite sequencing results by BSP. a GMS: Genome methylation sequencing.

TABLE S5 Strains and plasmids used in this study.

TABLE S6 Primers and oligonucleotide. Bold sequences are restriction sites. Where Y = C and T, R = A and G.
