# Supplementary figures and images for: Beyond a Ribosomal RNA Methyltransferase, the Wider Role of MraW in DNA Methylation, Motility and Colonization in Escherichia coli O157:H7
Source: Front Microbiol. 2019 Nov 13;10:2520. doi: 10.3389/fmicb.2019.02520 (PMC6863780; doi:10.3389/fmicb.2019.02520)

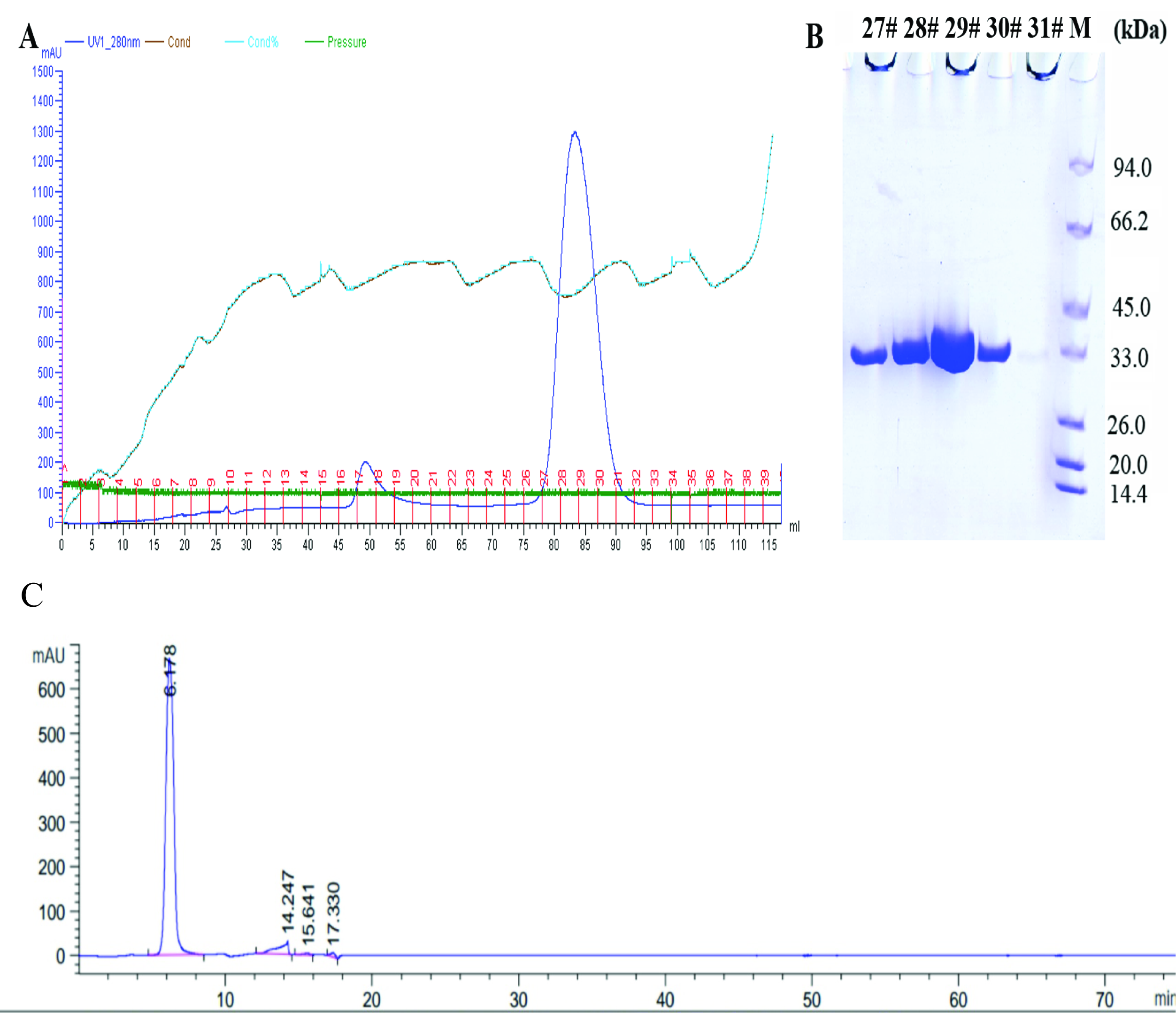

Supplement: FIGURE S1 — (A) MraW protein was purified and filtered by molecular sieve. MraW was first purified using gel filtration chromatogram (superdex 75). The single peak (78–90 ml) were collected and concentrated to 4 mg/ml. (B) The five tubes collected through molecular sieve were run on SDS-PAGE gel (27–31#). 27–31# is standing for the tube numbers from molecular sieve. MraW was indicated by black arrow. (C) The relative purity was 93.4% (peak area ratio) as determined by the HPLC-UV method. [file Image_1.tif]
