# Supplementary material for: Beyond a Ribosomal RNA Methyltransferase, the Wider Role of MraW in DNA Methylation, Motility and Colonization in Escherichia coli O157:H7
Source: Front Microbiol. 2019 Nov 13;10:2520. doi: 10.3389/fmicb.2019.02520 (PMC6863780; doi:10.3389/fmicb.2019.02520)
Supplement: TABLE S1 — Whole genome methylation level of C, CG, CHG, and CHH. H = A, T or C. [file Table_1.DOCX]

Table S1. Whole genome methylation level of C, CG, CHG and CHH

| Strain | C (%) | CG(%) | CHG(%) | CHH(%) |
| --- | --- | --- | --- | --- |
| EDL | 1.56 | 1.09 | 3.84 | 0.44 |
| EDL933ΔmraW | 1.42 | 0.96 | 3.50 | 0.41 |

H = A, T or C

Table S2 Promoter regions with DMRs between EDL and the ΔmraW

| Gene | Symbol | Methylation in EDL | Methylation in Δmraw | P-value | FDR | Description |
| --- | --- | --- | --- | --- | --- | --- |
| Cell envelope | |  |  |  |  |  |
| Z0290:- | fhiA | 0.008520768 | 0.004746751 | 0.00054166 | 0.0217891 | flagellar biosynthesis |
| Z3033:+ | fliK | 0.024125176 | 0.017199904 | 0.00098866 | 0.0288994 | flagellar hook-length control protein |
| Z3411:- | cirA | 0.012724513 | 0.004833269 | 0.00000005 | 0.0000188 | outer membrane receptor for iron-regulated colicin I receptor; porin; requires tonB gene product |
| Z5527:+ | btuB | 0.015037685 | 0.010341287 | 0.00114512 | 0.0319155 | outer membrane receptor for transport of vitamin B12, E colicins, and bacteriophage BF23 |
| Z4605:- | cafA | 0.012014114 | 0.009815557 | 0.00021188 | 0.0118352 | bundles of cytoplasmic filaments |
|  |  |  |  |  |  |  |
| Energy metabolism | |  |  |  |  |  |
| Z3197:- | fcI | 0.035103229 | 0.017875239 | 0.00002224 | 0.0025813 | fucose synthetase |
| Z5300:+ | rffH | 0.007877753 | 0.004249311 | 0.00000049 | 0.0001101 | glucose-1-phosphate thymidylyltransferase |
| Z4738:- | gph | 0.016281465 | 0.012666261 | 0.00094466 | 0.0288994 | phosphoglycolate phosphatase |
| Z4576:+ | gltB | 0.009815246 | 0.004214044 | 0.00013367 | 0.0081343 | glutamate synthase, large subunit |
| Z4796:- | glgB | 0.010053879 | 0.006014742 | 0.00000004 | 0.0000188 | 1,4-alpha-glucan branching enzyme |
| Z4239:+ | bglA | 0.016618621 | 0.011748906 | 0.00029704 | 0.0145115 | 6-phospho-beta-glucosidase A; cryptic |
| Z0750:+ | ahpF | 0.032801918 | 0.028654621 | 0.00023760 | 0.0121929 | alkyl hydroperoxide reductase, F52a subunit; detoxification of hydroperoxides |
| Z5250:+ | rbsA | 0.023311303 | 0.018203355 | 0.00000298 | 0.0004647 | ATP-binding component of D-ribose high-affinity transport system |
| Z3217:- | gmd | 0.021593378 | 0.016383787 | 0.00107976 | 0.0304407 | GDP-D-mannose dehydratase |
| Z5780:+ | hflX | 0.00820441 | 0.003661729 | 0.00000385 | 0.0005648 | GTP - binding subunit of protease specific for phage lambda cII repressor |
| Z3542:- | nuoG | 0.017298698 | 0.013344583 | 0.00002427 | 0.0027059 | NADH dehydrogenase I chain G |
| Z3534:- | nuoN | 0.032004302 | 0.024100943 | 0.00000290 | 0.0004647 | NADH dehydrogenase I chain N |
| Z4726:+ | nirB | 0.015000103 | 0.011305462 | 0.00091130 | 0.0287809 | nitrite reductase (NAD(P)H) subunit |
| Z5509:+ | frwD | 0.013445531 | 0.009056782 | 0.00012162 | 0.0078472 | PTS system fructose-like IIB component 2 |
| Z3190:- | ugd | 0.011505494 | 0.005123795 | 0.00151466 | 0.0383663 | UDP-glucose 6-dehydrogenase |
| Z4012:+ | srlD | 0.011905274 | 0.009405481 | 0.00089664 | 0.0287725 | glucitol (sorbitol)-6-phosphate dehydrogenase |
| Z5846:+ | cybC | 0.013340449 | 0.007453667 | 0.00012266 | 0.0078472 | cytochrome b (562) |
| Z4990:- | xylA | 0.00853163 | 0.004641328 | 0.00213860 | 0.0474294 | D-xylose isomerase |
| Z4094:- | eno | 0.017128309 | 0.018569042 | 0.00171646 | 0.0411785 | enolase |
| Z5436:- | fdoG | 0.018377234 | 0.01046793 | 0.00000009 | 0.0000291 | formate dehydrogenase-O, major subunit |
| Z5842:- | fbp | 0.020759037 | 0.018378352 | 0.00096826 | 0.0288994 | fructose-bisphosphatase |
| Z0089:+ | fruL | 0.009152291 | 0.003515692 | 0.00000000 | 0.0000022 | fruR leader peptide |
| Z3521:- | menC | 0.006573367 | 0.003856038 | 0.00008329 | 0.0063938 | o-succinylbenzoyl-CoA synthase; conversion of chorismate to 2-o-succinylbenzoyl-CoA |
| Z5675:+ | nrfG | 0.016129765 | 0.011119835 | 0.00000000 | 0.0000019 | part of formate-dependent nitrite reductase complex |
| Z4347:- | hybD | 0.02011551 | 0.017768276 | 0.00011012 | 0.0074420 | probable processing element for hydrogenase-2 |
| Z5579:+ | hydH | 0.013758621 | 0.011503863 | 0.00030798 | 0.0145115 | sensor kinase for HydG, hydrogenase 3 activity |
| Z3810:+ | hcaA2 | 0.027340332 | 0.022902806 | 0.00031926 | 0.0145115 | small terminal subunit of phenylpropionate dioxygenase |
| Z4729:+ | cysG | 0.023198283 | 0.018979985 | 0.00002549 | 0.0027059 | uroporphyrinogen III methylase; sirohaeme biosynthesis |
| Z5318:- | hemD | 0.005101024 | 0.003789973 | 0.00161798 | 0.0395771 | uroporphyrinogen III synthase |
|  |  |  |  |  |  |  |
| Environmental, metabolic response | | |  |  |  |  |
| Z0204:+ | cutF | 0.023640773 | 0.017259452 | 0.00000102 | 0.0001954 | copper homeostasis protein (lipoprotein) |
| Z3712:- | cchB | 0.024888281 | 0.018838093 | 0.00226980 | 0.0496768 | detox protein |
| Z4661:+ | mscL | 0.02446261 | 0.019303162 | 0.00006979 | 0.0055275 | mechanosensitive channel |
| Z0063:- | imp | 0.008692617 | 0.004296797 | 0.00056154 | 0.0220637 | organic solvent tolerance |
| Z0031:+ | lspA | 0.016303202 | 0.013514689 | 0.00219720 | 0.0485134 | prolipoprotein signal peptidase (SPase II) |
| Z5714:- | basS | 0.012830088 | 0.009701927 | 0.00013156 | 0.0081343 | sensor protein for basR |
|  |  |  |  |  |  |  |
| Nucleotide metabolism | |  |  |  |  |  |
| Z5984:+ | deoA | 0.004605808 | 0.003044829 | 0.00093854 | 0.0288994 | thymidine phosphorylase |
|  |  |  |  |  |  |  |
| Amino acid metabolism | | |  |  |  |  |
| Z3233:- | dcd | 0.017335612 | 0.010907836 | 0.00055236 | 0.0218752 | 2'-deoxycytidine 5'-triphosphate deaminase |
| Z5057:+ | kdtA | 0.01764902 | 0.010899603 | 0.00023586 | 0.0121929 | 3-deoxy-D-manno-octulosonic-acid transferase (KDO transferase) |
| Z0298:- | pepD | 0.011128342 | 0.007821298 | 0.00023704 | 0.0121929 | aminoacyl-histidine dipeptidase (peptidase D) |
| Z5283:+ | ilvA | 0.03064686 | 0.02777725 | 0.00060312 | 0.0226283 | threonine deaminase (dehydratase) |
| Z4471:+ | tdcR | 0.036108972 | 0.023031026 | 0.00085282 | 0.0281586 | threonine dehydratase operon activator protein |
| Z0102:+ | ddlB | 0.013266871 | 0.011001407 | 0.00091826 | 0.0288183 | D-alanine-D-alanine ligase B, affects cell division |
| Z3870:+ | pssA | 0.008292603 | 0.003524778 | 0.00021942 | 0.0120319 | phosphatidylserine synthase; phospholipid synthesis |
|  |  |  |  |  |  |  |
| Central intermediary metabolism | | |  |  |  |  |
| Z5355:+ | ubiE | 0.018385308 | 0.014376685 | 0.00001496 | 0.0018667 | 2-octaprenyl-6-methoxy-1,4-benzoquinone --> 2-octaprenyl-3-methyl-6-methoxy-1,4-benzoquinone |
| Z0033:+ | slpA | 0.017006198 | 0.012459788 | 0.00009210 | 0.0066604 | probable FKBX-type 16KD peptidyl-prolyl cis-trans isomerase (a rotamase) |
|  |  |  |  |  |  |  |
| Replication |  |  |  |  |  |  |
| Z0589:+ | recR | 0.022761461 | 0.01645198 | 0.00101584 | 0.0293008 | recombination and repair |
| Z0787:- | holA | 0.028547639 | 0.025705542 | 0.00030950 | 0.0145115 | DNA polymerase III, delta subunit |
| Z3909:+ | recN | 0.023146266 | 0.018862354 | 0.00000484 | 0.0006557 | protein used in recombination and DNA repair |
| Z0865:+ | nei | 0.013657925 | 0.010967718 | 0.00156918 | 0.0392189 | endonuclease VIII and DNA N-glycosylase with an AP lyase activity |
| Z5328:+ | xerC | 0.011023213 | 0.00880307 | 0.00018145 | 0.0105281 | site-specific recombinase, acts on cer sequence of ColE1, effects chromosome segregation at cell division |
|  |  |  |  |  |  |  |
| Transcription | |  |  |  |  |  |
| Z4565:+ | rpoN | 0.004789777 | 0.003198677 | 0.00089678 | 0.0287725 | RNA polymerase, sigma(54 or 60) factor; nitrogen and fermentation regulation |
|  |  |  |  |  |  |  |
| Translation |  |  |  |  |  |  |
| Z4658:+ | fmt | 0.034210477 | 0.028520921 | 0.00097860 | 0.0288994 | 10-formyltetrahydrofolate:L-methionyl-tRNA(fMet) N-formyltransferase |
| Z4685:- | rpsC | 0.010982999 | 0.006177692 | 0.00000023 | 0.0000651 | 30S ribosomal subunit protein S3 |
| Z3900:- | rplS | 0.032731724 | 0.026898591 | 0.00000074 | 0.0001534 | 50S ribosomal subunit protein L19 |
| Z4669:- | rpmJ | 0.031277387 | 0.026179627 | 0.00022426 | 0.0120329 | 50S ribosomal subunit protein L36 |
| Z4678:- | rplE | 0.01319255 | 0.011932653 | 0.00078874 | 0.0269576 | 50S ribosomal subunit protein L5 |
| Z5526:- | trmA | 0.007758957 | 0.004329902 | 0.00104932 | 0.0297506 | tRNA (uracil-5-)-methyltransferase |
| Z0206:- | proS | 0.01702121 | 0.011932209 | 0.00160102 | 0.0395499 | proline tRNA synthetase |
| Z5011:- | selB | 0.01230548 | 0.010675018 | 0.00103732 | 0.0296560 | selenocysteinyl-tRNA-specific translation factor |
| Z4697:- | tufA | 0.005313972 | 0.003317183 | 0.00068796 | 0.0245076 | protein chain elongation factor EF-Tu (duplicate of tufB) |
|  |  |  |  |  |  |  |
| Transport and binding protein | | |  |  |  |  |
| Z5081:- | gltS | 0.021794065 | 0.016443869 | 0.00079466 | 0.0269752 | glutamate transport |
| Z5676:+ | gltP | 0.018438686 | 0.010883808 | 0.00115126 | 0.0319155 | glutamate-aspartate symport protein |
| Z4618:+ | panF | 0.007506136 | 0.004239079 | 0.00000010 | 0.0000309 | sodium/pantothenate symporter |
| Z4552:- | murA | 0.018528952 | 0.013979882 | 0.00003402 | 0.0032648 | first step in murein biosynthesis;UDP-N-glucosamine 1-carboxyvinyltransferase |
| Z4288:+ | galP | 0.015906668 | 0.002867782 | 0.00000184 | 0.0003394 | galactose-proton symport of transport system |
| Z0933:+ | modA | 0.025342054 | 0.018462918 | 0.00024870 | 0.0123505 | molybdate-binding periplasmic protein; permease |
| Z5631:- | malF | 0.018789479 | 0.016539332 | 0.00097404 | 0.0288994 | part of maltose permease, periplasmic |
|  |  |  |  |  |  |  |
| Biosynthesis of cofactors, prosthetic groups, carriers | | | |  |  |  |
| Z1254:+ | aroA | 0.015416097 | 0.011082814 | 0.00052650 | 0.0215347 | 5-enolpyruvylshikimate-3-phosphate synthetase |
| Z0142:- | panD | 0.005914385 | 0.003524974 | 0.00015095 | 0.0089670 | aspartate 1-decarboxylase |
| Z3452:- | dsbE | 0.011339406 | 0.007949889 | 0.00086338 | 0.0281586 | disulfide oxidoreductase (in biogenesis of cytochrome c |
|  |  |  |  |  |  |  |
| Regulatory functions | |  |  |  |  |  |
| Z5855:- | pyrI | 0.020550587 | 0.016863598 | 0.00024794 | 0.0123505 | aspartate carbamoyltransferase, regulatory subunit |
| Z4602:+ | qseA | 0.020763306 | 0.01659783 | 0.00193366 | 0.0447594 | quorum sensing Escherichia coli regulator A |
| Z4377:+ | qseB | 0.021779119 | 0.018398696 | 0.00008714 | 0.0064946 | quorum sensing Escherichia coli regulator B |
| Z5025:+ | mtlR | 0.016892098 | 0.0142268 | 0.00012454 | 0.0078665 | repressor for mtl |
| Z4781:- | glpR | 0.013324197 | 0.011947381 | 0.00093854 | 0.0288994 | repressor of the glp operon |
| Z5851:- | treR | 0.011996456 | 0.008551194 | 0.00031250 | 0.0145115 | repressor of treA,B,C |
| Z3146:- | cbl | 0.00986613 | 0.005042996 | 0.00130328 | 0.0345924 | transcriptional regulator cys regulon; accessory regulatory circuit affecting cysM |
| Z0301:+ | crl | 0.004517959 | 0.003053699 | 0.00157976 | 0.0392189 | transcriptional regulator of cryptic csgA gene for curli surface fibers |
| Z5715:- | basR | 0.021451908 | 0.019096528 | 0.00063520 | 0.0230422 | transcriptional regulatory protein 2-component regulatory system member |
| Z0090:+ | fruR | 0.018158708 | 0.012109903 | 0.00000001 | 0.0000083 | transcriptional repressor of fru operon and others |
| Z3888:- | sfhB | 0.008129971 | 0.004380387 | 0.00013937 | 0.0083789 | suppressor of ftsH mutation |
| Z4013:+ | gutM | 0.005092325 | 0.003216572 | 0.00063724 | 0.0230422 | glucitol operon activator |
| Z5450:+ | rhaR | 0.025157601 | 0.022056465 | 0.00193748 | 0.0447594 | positive regulator for rhaRS operon |
| Z0931:- | modE | 0.02497954 | 0.018126192 | 0.00045856 | 0.0191100 | molybdate uptake regulatory protein |
|  |  |  |  |  |  |  |
| Other categories | |  |  |  |  |  |
| Z3093:+ |  | 0.031081679 | 0.022189121 | 0.00000006 | 0.0000210 | unknown in IS629 encoded within prophage CP-933U |
| Z0957:+ |  | 0.012334536 | 0.003793216 | 0.00000040 | 0.0000943 | unknown protein encoded by prophage CP-933K |
| Z0949:- |  | 0.011904695 | 0.008328383 | 0.00193152 | 0.0447594 | unknown protein encoded by prophage CP-933K |
| Z2051:+ |  | 0.020610001 | 0.015889741 | 0.00029972 | 0.0145115 | unknown protein encoded by prophage CP-933O |
| Z2100:+ |  | 0.019449659 | 0.013223706 | 0.00195744 | 0.0450121 | unknown protein encoded within prophage CP-933O |
| Z3127:+ |  | 0.02008541 | 0.011317769 | 0.00005219 | 0.0045237 | unknown protein encoded within prophage CP-933U |
| Z5781:+ | hflK | 0.008992146 | 0.006074861 | 0.00001133 | 0.0014492 | protease specific for phage lambda cII repressor |
| Z5253:+ | rbsK | 0.006033773 | 0.003704133 | 0.00154222 | 0.0388671 | ribokinase |
| Z0748:- | dsbG | 0.035789388 | 0.023263221 | 0.00021346 | 0.0118352 | thiol:disulfide interchange protein |

Table S3 Genes with DMRs between EDL and the ΔmraW

| Gene | Symbol | Methylation in EDL | Methylation in Δmraw | P-value | FDR | Description |
| --- | --- | --- | --- | --- | --- | --- |
| Cell envelope |  |  |  |  |  |  |
| Z3040:+ | fliR | 0.012005387 | 0.00904308 | 0.0001940 | 0.0067149 | flagellar biosynthesis |
| Z3032:+ | fliJ | 0.012938192 | 0.008469078 | 0.0010508 | 0.0214588 | flagellar fliJ protein |
| Z0301:+ | crl | 0.007057367 | 0.002349398 | 0.0005195 | 0.0134531 | transcriptional regulator of cryptic csgA gene for curli surface fibers |
| Z2942:- | cheA | 0.016880508 | 0.01469936 | 0.0025216 | 0.0392660 | sensory transducer kinase between chemo- signal receptors and CheB and CheY |
| Z5632:- | malE | 0.025538994 | 0.023444678 | 0.0020144 | 0.0336751 | periplasmic maltose-binding protein; substrate recognition for transport and chemotaxis |
| Z5756:- | blc | 0.012617067 | 0.010293913 | 0.0004304 | 0.0117862 | outer membrane lipoprotein (lipocalin) |
| Z5342:+ | pldA | 0.017573444 | 0.01181132 | 0.0000000 | 0.0000026 | outer membrane phospholipase A |
| Z5109:+ | escD | 0.025204849 | 0.016871733 | 0.0000001 | 0.0000307 | escD |
| Z5105:- | espB | 0.009737305 | 0.007432959 | 0.0000883 | 0.0038134 | secreted protein EspB |
| Z4187:- |  | 0.015028577 | 0.004116929 | 0.0000000 | 0.0000005 | type III secretion apparatus protein |
|  |  |  |  |  |  |  |
| Energy metabolism | |  |  |  |  |  |
| Z4583:- | nanA | 0.018126626 | 0.014701524 | 0.0000019 | 0.0002047 | N-acetylneuraminate lyase (aldolase); catabolism of sialic acid; not K-12 |
| Z5249:+ | rbsD | 0.02635989 | 0.020589471 | 0.0000031 | 0.0003012 | D-ribose high-affinity transport system; membrane-associated protein |
| Z0647:- | tesA | 0.009164956 | 0.005905392 | 0.0028722 | 0.0432392 | acyl-CoA thioesterase I; also functions as protease I |
| Z4254:+ | sbm | 0.01985124 | 0.017618686 | 0.0000765 | 0.0034046 | methylmalonyl-CoA mutase (MCM) |
| Z0749:+ | ahpC | 0.040785184 | 0.030534078 | 0.0000062 | 0.0005025 | alkyl hydroperoxide reductase, C22 subunit; detoxification of hydroperoxides |
| Z5743:- | dcuA | 0.015874998 | 0.014143486 | 0.0002857 | 0.0088419 | anaerobic dicarboxylate transport |
| Z0873:- | gltA | 0.012886542 | 0.010411856 | 0.0024412 | 0.0382531 | citrate synthase |
| Z3701:+ | hemF | 0.012997793 | 0.011123484 | 0.0028204 | 0.0427175 | coproporphyrinogen III oxidase |
| Z4156:- | lysA | 0.015853319 | 0.013551765 | 0.0009585 | 0.0201532 | diaminopimelate decarboxylase |
| Z5282:+ | ilvD | 0.018921185 | 0.016328755 | 0.0000641 | 0.0030147 | dihydroxyacid dehydratase |
| Z3522:- | menB | 0.009803589 | 0.007800906 | 0.0003224 | 0.0094187 | dihydroxynaphtoic acid synthetase |
| Z3382:+ | dld | 0.010027836 | 0.008401482 | 0.0012217 | 0.0239682 | D-lactate dehydrogenase, FAD protein, NADH independent |
| Z4739:- | rpe | 0.023554524 | 0.019682549 | 0.0013627 | 0.0254312 | D-ribulose-5-phosphate 3-epimerase |
| Z4429:+ | ebgA | 0.014982339 | 0.013307239 | 0.0001656 | 0.0059792 | evolved beta-D-galactosidase, alpha subunit; cryptic gene |
| Z5678:- | fdhF | 0.020335028 | 0.017450386 | 0.0001485 | 0.0055231 | formate dehydrogenase |
| Z5434:- | fdoI | 0.016609061 | 0.012923369 | 0.0003232 | 0.0094187 | formate dehydrogenase, cytochrome B556 (FDO) subunit |
| Z2236:- | fdnG | 0.015162009 | 0.012948336 | 0.0007741 | 0.0174225 | formate dehydrogenase-N, nitrate-inducible, alpha subunit |
| Z5842:- | fbp | 0.023348307 | 0.020214901 | 0.0000126 | 0.0008406 | fructose-bisphosphatase |
| Z4012:+ | srlD | 0.005413626 | 0.004318844 | 0.0012493 | 0.0242232 | glucitol (sorbitol)-6-phosphate dehydrogenase |
| Z4790:- | glgP | 0.023260997 | 0.020691797 | 0.0000000 | 0.0000096 | glycogen phosphorylase |
| Z4791:- | glgA | 0.012544148 | 0.010681139 | 0.0008358 | 0.0180911 | glycogen synthase |
| Z5747:+ | mopB | 0.006089612 | 0.003126658 | 0.0001059 | 0.0043266 | GroES, 10 Kd chaperone binds to Hsp60 in pres. Mg-ATP, suppressing its ATPase activity |
| Z5780:+ | hflX | 0.025630464 | 0.021022466 | 0.0000000 | 0.0000003 | GTP - binding subunit of protease specific for phage lambda cII repressor |
| Z4698:- | fusA | 0.020947924 | 0.019623859 | 0.0006141 | 0.0150913 | GTP-binding protein chain elongation factor EF-G |
| Z5030:+ | lldP | 0.01592374 | 0.014314721 | 0.0002255 | 0.0074338 | L-lactate permease |
| Z3543:- | nuoF | 0.021226045 | 0.016798959 | 0.0000000 | 0.0000002 | NADH dehydrogenase I chain F |
| Z4728:+ | nirC | 0.023529619 | 0.018983801 | 0.0000301 | 0.0017016 | nitrite reductase |
| Z3727:+ | acrD | 0.015305267 | 0.012357445 | 0.0001912 | 0.0066622 | sensitivity to acriflavine, integral membrane protein, possible efflux pump |
| Z4743:- | aroK | 0.008899385 | 0.006091302 | 0.0023812 | 0.0376683 | shikimate kinase I |
| Z0599:+ | ushA | 0.012346579 | 0.009623855 | 0.0001190 | 0.0046495 | UDP-sugar hydrolase (5'-nucleotidase) |
| Z5569:- | thiC | 0.022512289 | 0.019254113 | 0.0002248 | 0.0074338 | thiamin biosynthesis, pyrimidine moiety |
| Z4989:- | xylB | 0.006529797 | 0.005661516 | 0.0013183 | 0.0250732 | xylulokinase |
| Z3813:+ | hcaB | 0.013150423 | 0.011432431 | 0.0030052 | 0.0447012 | 2,3-dihydroxy-2,3-dihydrophenylpropionate dehydrogenase |
| Z4577:+ | gltD | 0.013241623 | 0.011860098 | 0.0030494 | 0.0450895 | glutamate synthase, small subunit |
| Z5676:+ | gltP | 0.018030435 | 0.016377718 | 0.0004117 | 0.0113972 | glutamate-aspartate symport protein |
| Z4292:+ | gshB | 0.015145161 | 0.012380685 | 0.0001645 | 0.0059792 | glutathione synthetase |
| Z5674:+ | nrfF | 0.020327816 | 0.013486334 | 0.0000000 | 0.0000000 | part of formate-dependent nitrite reductase complex |
| Z0088:+ | ilvH | 0.009131228 | 0.003515778 | 0.0000000 | 0.0000015 | acetolactate synthase III, valine sensitive, small subunit |
| Z3682:+ | ptsI | 0.010896671 | 0.009281991 | 0.0022260 | 0.0363677 | PEP-protein phosphotransferase system enzyme I |
|  |  |  |  |  |  |  |
| Environmental, metabolic response | | |  |  |  |  |
| Z5563:+ | htrC | 0.005808694 | 0.003247485 | 0.0011444 | 0.0228094 | heat shock protein C |
| Z4052:- | surE | 0.011447004 | 0.007563565 | 0.0029670 | 0.0443981 | survival protein |
| Z0788:- | rlpB | 0.024702878 | 0.022444508 | 0.0014035 | 0.0257115 | a minor lipoprotein |
| Z0031:+ | lspA | 0.019057753 | 0.012653152 | 0.0000002 | 0.0000378 | prolipoprotein signal peptidase (SPase II) |
| Z4540:- | hflB | 0.029692729 | 0.027760439 | 0.0022536 | 0.0366983 | degrades sigma32, integral membrane peptidase, cell division protein |
| Z0063:- | imp | 0.020857338 | 0.018781539 | 0.0023644 | 0.0375217 | organic solvent tolerance |
| Z6003:+ | creD | 0.005512078 | 0.003627026 | 0.0018560 | 0.0318707 | tolerance to colicin E2 |
|  |  |  |  |  |  |  |
| Nucleotide metabolism | |  |  |  |  |  |
| Z0109:+ | mutT | 0.009050476 | 0.004423836 | 0.0027742 | 0.0421458 | 7,8-dihydro-8-oxoguanine-triphosphatase, prefers dGTP, causes AT-GC transversions |
| Z2197:+ | hipA | 0.022360952 | 0.015779985 | 0.0005211 | 0.0134531 | persistence to inhibition of murein or DNA biosynthesis, DNA-binding regulator |
| Z0154:- | pcnB | 0.016574837 | 0.014453665 | 0.0000162 | 0.0010109 | poly(A) polymerase I |
| Z3580:- | truA | 0.01271437 | 0.009666056 | 0.0009929 | 0.0206674 | pseudouridylate synthase I |
| Z3977:+ | nrdE | 0.009472561 | 0.007746443 | 0.0012720 | 0.0244002 | ribonucleoside-diphosphate reductase 2, alpha subunit |
| Z5318:- | hemD | 0.01873358 | 0.016403508 | 0.0032310 | 0.0469390 | uroporphyrinogen III synthase |
|  |  |  |  |  |  |  |
| Amino acid metabolism | |  |  |  |  |  |
| Z4997:+ | avtA | 0.025797081 | 0.020595822 | 0.0000131 | 0.0008500 | alanine-alpha-ketoisovalerate (or valine-pyruvate) transaminase, transaminase C |
| Z0122:- | aroP | 0.018637602 | 0.015089884 | 0.0000012 | 0.0001585 | aromatic amino acid transport protein |
| Z0002:+ | thrA | 0.017707888 | 0.01561062 | 0.0001835 | 0.0064382 | aspartokinase I, homoserine dehydrogenase I |
| Z3686:- | cysM | 0.01384449 | 0.008906533 | 0.0004906 | 0.0128797 | cysteine synthase B, O-acetylserine sulfhydrolase B |
| Z4544:+ | dacB | 0.021577505 | 0.019363857 | 0.0000561 | 0.0027426 | D-alanyl-D-alanine carboxypeptidase, fraction B; penicillin-binding protein 4 |
| Z3765:+ | ppx | 0.017744922 | 0.014844558 | 0.0002751 | 0.0086767 | exopolyphosphatase |
| Z5405:- | glnL | 0.030975139 | 0.028158862 | 0.0022942 | 0.0371169 | histidine protein kinase sensor for GlnG regulator (nitrogen regulator II, NRII) |
| Z0101:+ | murC | 0.019334689 | 0.017084323 | 0.0000017 | 0.0001989 | L-alanine adding enzyme, UDP-N-acetyl-muramate:alanine ligase |
| Z5204:+ | tnaB | 0.018398173 | 0.016231263 | 0.0000064 | 0.0005083 | low affinity tryptophan permease |
| Z4310:- | speC | 0.0152172 | 0.013468034 | 0.0003070 | 0.0092708 | ornithine decarboxylase isozyme |
| Z5976:+ | prfC | 0.020144201 | 0.016697226 | 0.0003295 | 0.0095453 | peptide chain release factor RF-3 |
| Z4657:+ | def | 0.033901891 | 0.028442285 | 0.0023300 | 0.0375206 | peptide deformylase |
| Z4529:- | infB | 0.019609179 | 0.01819071 | 0.0012049 | 0.0237309 | protein chain initiation factor IF-2 |
| Z0125:+ | aceF | 0.01073603 | 0.008872282 | 0.0000550 | 0.0027224 | pyruvate dehydrogenase (dihydrolipoyltransacetylase component) |
| Z4593:+ | degQ | 0.016772253 | 0.013680286 | 0.0000005 | 0.0000957 | serine endoprotease |
| Z3720:+ | talA | 0.020912916 | 0.015639317 | 0.0014003 | 0.0257115 | transaldolase A |
|  |  |  |  |  |  |  |
| Central intermediary metabolism | | |  |  |  |  |
| Z4372:- | plsC | 0.005368561 | 0.003139366 | 0.0002846 | 0.0088419 | 1-acyl-sn-glycerol-3-phosphate acyltransferase |
| Z0081:- | leuC | 0.026619607 | 0.024606893 | 0.0020274 | 0.0336751 | 3-isopropylmalate isomerase (dehydratase) subunit |
| Z5299:+ | rffG | 0.013879546 | 0.010179457 | 0.0007268 | 0.0165363 | dTDP-glucose 4,6-dehydratase |
| Z4990:- | xylA | 0.01715037 | 0.014307556 | 0.0006082 | 0.0150858 | D-xylose isomerase |
| Z5472:- | glpF | 0.017769062 | 0.0129636 | 0.0000036 | 0.0003254 | facilitated diffusion of glycerol |
| Z4940:+ | kdgK | 0.017007157 | 0.013330028 | 0.0000000 | 0.0000003 | ketodeoxygluconokinase |
| Z4116:- | fucO | 0.014893927 | 0.012181864 | 0.0000184 | 0.0010915 | L-1,2-propanediol oxidoreductase |
| Z4595:- | mdh | 0.026693952 | 0.02372758 | 0.0007058 | 0.0163424 | malate dehydrogenase |
| Z5024:+ | mtlD | 0.01500589 | 0.012563391 | 0.0000035 | 0.0003220 | mannitol-1-phosphate dehydrogenase |
| Z5853:+ | mgtA | 0.00815649 | 0.006521103 | 0.0000021 | 0.0002173 | Mg2+ transport ATPase, P-type 1 |
| Z4784:- | glpG | 0.011827035 | 0.010375716 | 0.0009322 | 0.0197670 | protein of glp regulon |
|  |  |  |  |  |  |  |
| Replication |  |  |  |  |  |  |
| Z5962:- | dnaT | 0.030964664 | 0.027605694 | 0.0035182 | 0.0495231 | DNA biosynthesis; primosomal protein i |
| Z5190:- | gyrB | 0.013774356 | 0.011653771 | 0.0019359 | 0.0325549 | DNA gyrase subunit B, type II topoisomerase, ATPase activity |
| Z3484:- | gyrA | 0.019866996 | 0.016425528 | 0.0000156 | 0.0009834 | DNA gyrase, subunit A, type II topoisomerase |
| Z4136:- | recD | 0.010856924 | 0.008825324 | 0.0001038 | 0.0042734 | DNA helicase, ATP-dependent dsDNA/ssDNA exonuclease V subunit, ssDNA endonuclease |
| Z3677:- | lig | 0.010398417 | 0.008152985 | 0.0002171 | 0.0072598 | DNA ligase |
| Z5070:+ | dinD | 0.020383757 | 0.016282124 | 0.0000007 | 0.0001105 | DNA-damage-inducible protein |
|  |  |  |  |  |  |  |
| Transcription |  |  |  |  |  |  |
| Z4658:+ | fmt | 0.011827757 | 0.010013201 | 0.0000963 | 0.0040823 | 10-formyltetrahydrofolate:L-methionyl-tRNA(fMet) N-formyltransferase |
| Z0681:+ | cysS | 0.012199931 | 0.009969167 | 0.0012006 | 0.0237309 | cysteine tRNA synthetase |
| Z5561:+ | rpoC | 0.019898172 | 0.017772171 | 0.0015854 | 0.0282152 | RNA polymerase, beta prime subunit |
| Z5012:- | selA | 0.008106362 | 0.006728031 | 0.0001639 | 0.0059792 | selenocysteine synthase: L-seryl-tRNA (Ser) selenium transferase |
| Z3901:- | trmD | 0.019594861 | 0.015480267 | 0.0001069 | 0.0043315 | tRNA methyltransferase; tRNA (guanine-7-)-methyltransferase |
| Z2313:- | hrpA | 0.015911925 | 0.013617534 | 0.0025944 | 0.0401952 | helicase, ATP-dependent |
| Z0159:+ | hrpB | 0.021546204 | 0.019510244 | 0.0006085 | 0.0150858 | helicase, ATP-dependent |
| Z4470:- | tdcA | 0.018405673 | 0.011962695 | 0.0000108 | 0.0007610 | transcriptional activator of tdc operon |
| Z4020:- | hypF | 0.010544942 | 0.008949504 | 0.0003548 | 0.0101613 | transcriptional regulatory protein |
| Z5715:- | basR | 0.015846777 | 0.01295937 | 0.0003206 | 0.0094187 | transcriptional regulatory protein 2-component regulatory system member |
|  |  |  |  |  |  |  |
| Translation |  |  |  |  |  |  |
| Z0180:+ | rpsB | 0.028845746 | 0.025223889 | 0.0018254 | 0.0314739 | 30S ribosomal subunit protein S2 |
| Z4588:- | rpsI | 0.010227068 | 0.00593412 | 0.0001330 | 0.0050981 | 30S ribosomal subunit protein S9 |
| Z4686:- | rplV | 0.009018365 | 0.006237519 | 0.0000658 | 0.0030636 | 50S ribosomal subunit protein L22 |
| Z1079:+ | rimK | 0.012415812 | 0.009201878 | 0.0002053 | 0.0069122 | ribosomal protein S6 modification protein |
|  |  |  |  |  |  |  |
| Transport and binding protein | | |  |  |  |  |
| Z4771:- | malQ | 0.015370591 | 0.013167962 | 0.0000015 | 0.0001886 | 4-alpha-glucanotransferase (amylomaltase) |
| Z3441:- | bcr | 0.008737607 | 0.004981139 | 0.0000001 | 0.0000213 | bicyclomycin resistance protein; transmembrane protein |
| Z0494:- | araJ | 0.013755624 | 0.009329633 | 0.0013244 | 0.0250939 | involved in either transport or processing of arabinose polymers |
| Z0673:+ | fdrA | 0.016644703 | 0.013820383 | 0.0000636 | 0.0030147 | involved in protein transport; multicopy suppressor of dominant negative ftsH mutants |
| Z4772:- | malP | 0.011558123 | 0.009841432 | 0.0000001 | 0.0000160 | maltodextrin phosphorylase |
| Z4004:- | mltB | 0.019008922 | 0.015405565 | 0.0000499 | 0.0025349 | membrane-bound lytic murein transglycosylase B |
| Z4030:- | hycD | 0.015713262 | 0.013365092 | 0.0013847 | 0.0255547 | membrane-spanning protein of hydrogenase 3 (part of FHL complex) |
| Z4341:- | pitB | 0.020920146 | 0.018011164 | 0.0006299 | 0.0150913 | partial putative transport protein |
| Z3659:+ | nupC | 0.023814898 | 0.021455689 | 0.0001208 | 0.0046648 | permease of transport system for 3 nucleosides |
| Z0108:+ | secA | 0.017734483 | 0.01605819 | 0.0030292 | 0.0449241 | preprotein translocase; secretion protein |
| Z4537:- | secG | 0.006997632 | 0.004020357 | 0.0000637 | 0.0030147 | protein export - membrane protein |
| Z4146:- | ptsP | 0.016941505 | 0.014803248 | 0.0004491 | 0.0121796 | PTS system, enzyme I, transcriptional regulator (with NPR and NTR proteins) |
| Z3425:- | fruA | 0.014064143 | 0.011773815 | 0.0013604 | 0.0254312 | PTS system, fructose-specific transport protein |
| Z4011:+ | srlB | 0.005240597 | 0.003052511 | 0.0028448 | 0.0429565 | PTS system, glucitol/sorbitol-specific enzyme IIA component |
| Z5023:+ | mtlA | 0.016798457 | 0.014249211 | 0.0000079 | 0.0006163 | PTS system, mannitol-specific enzyme IIABC components |
| Z5999:- | rob | 0.016212435 | 0.013577368 | 0.0000188 | 0.0011001 | right origin-binding protein |
| Z4618:+ | panF | 0.013109633 | 0.01123456 | 0.0000042 | 0.0003711 | sodium/pantothenate symporter |
| Z1830:- | potB | 0.014643854 | 0.009643933 | 0.0000883 | 0.0038134 | spermidine/putrescine transport system permease |
| Z5735:- | cadB | 0.016472273 | 0.014322075 | 0.0022102 | 0.0362284 | transport of lysine/cadaverine |
|  |  |  |  |  |  |  |
| Biosynthesis of cofactors, prosthetic groups, carriers | | | |  |  |  |
| Z5316:- | hemY | 0.00909102 | 0.00670093 | 0.0000413 | 0.0022140 | a late step of protoheme IX synthesis |
| Z5856:- | pyrB | 0.021038628 | 0.017004476 | 0.0000058 | 0.0004785 | aspartate carbamoyltransferase, catalytic subunit |
| Z0515:+ | ribD | 0.013777433 | 0.010398999 | 0.0021494 | 0.0354651 | bifunctional pyrimidine deaminase/reductase in pathway of riboflavin synthesis |
| Z4399:- | ribB | 0.016688983 | 0.014437435 | 0.0025974 | 0.0401952 | 3,4 dihydroxy-2-butanone-4-phosphate synthase |
| Z0038:+ | carB | 0.014509171 | 0.013243545 | 0.0002896 | 0.0089073 | carbamoyl-phosphate synthase large subunit |
| Z5388:- | mobB | 0.023117526 | 0.0189916 | 0.0000182 | 0.0010915 | molybdopterin-guanine dinucleotide biosynthesis protein B |
| Z0041:- | caiE | 0.007330775 | 0.003871701 | 0.0000000 | 0.0000027 | possible synthesis of cofactor for carnitine racemase and dehydratase |
|  |  |  |  |  |  |  |
| Regulatory functions | |  |  |  |  |  |
| Z5519:+ | oxyR | 0.02128891 | 0.018587128 | 0.0031000 | 0.0457021 | activator, hydrogen peroxide-inducible genes |
| Z5215:- | phoU | 0.017469362 | 0.014198776 | 0.0000026 | 0.0002545 | negative regulator for pho regulon and putative enzyme in phosphate metabolism |
| Z5910:+ | fimB | 0.012880429 | 0.010450116 | 0.0007241 | 0.0165363 | recombinase involved in phase variation; regulator for fimA |
| Z5609:- | iclR | 0.009145849 | 0.007864169 | 0.0010043 | 0.0207661 | repressor of aceBA operon |
| Z4101:+ | barA | 0.019907731 | 0.017187785 | 0.0000120 | 0.0008310 | sensor-regulator, activates OmpR by phophorylation |
| Z4603:- | tldD | 0.017036322 | 0.01482299 | 0.0003982 | 0.0111479 | suppresses inhibitory activity of CsrA |
|  |  |  |  |  |  |  |
| Other categories | |  |  |  |  |  |
| Z5779:+ | hfq | 0.00754293 | 0.004244142 | 0.0016973 | 0.0298853 | host factor I for bacteriophage Q beta replication, a growth-related protein |
| Z3907:- | grpE | 0.019917144 | 0.015357067 | 0.0006295 | 0.0150913 | phage lambda replication; host DNA synthesis; heat shock protein; protein repair |
| Z1359:+ | Z1359:+ | 0.027981551 | 0.01715808 | 0.0015216 | 0.0274722 | unknown protein encoded by cryptic prophage CP-933M |
| Z0950:- | Z0950:- | 0.023992695 | 0.018203194 | 0.0008210 | 0.0179497 | unknown protein encoded by prophage CP-933K |
| Z2049:+ | Z2049:+ | 0.027376391 | 0.020883802 | 0.0000002 | 0.0000449 | unknown protein encoded by prophage CP-933O |
| Z2056:+ | Z2056:+ | 0.018072911 | 0.012085379 | 0.0000030 | 0.0002944 | unknown protein encoded by prophage CP-933O |
| Z2975:+ | Z2975:+ | 0.017409187 | 0.013563029 | 0.0027592 | 0.0420462 | unknown protein encoded by prophage CP-933T |
| Z5091:+ | Z5091:+ | 0.023185651 | 0.018465004 | 0.0032862 | 0.0474642 | unknown protein encoded within prophage CP-933L |
| Z3092:- | Z3092:- | 0.026417788 | 0.022774495 | 0.0010228 | 0.0209741 | unknown protein encoded within prophage CP-933U |
| Z2540:+ | btuR | 0.034199122 | 0.02317179 | 0.0000044 | 0.0003808 | cob(I)alamin adenolsyltransferase |
| Z3713:- | cchA | 0.034438441 | 0.025703861 | 0.0001675 | 0.0060045 | detox protein |

Table S4 Validation of bisulfite sequencing results by BSP

|  |  |  |  |
| --- | --- | --- | --- |
|  |  | Methylation in EDL | Methylation in ΔmraW |
| fliR | GMS | 0.0120 | 0.0090 |
|  | BSP | 0.0253 | 0.0164 |
|  |  |  |  |
| fliJ | GMS | 0.0129 | 0.0085 |
|  | BSP | 0.0255 | 0.0169 |
|  |  |  |  |
| fliK | GMS | 0.0241 | 0.0172 |
|  | BSP | 0.0502 | 0.0334 |
|  |  |  |  |
| fhiA | GMS | 0.0085 | 0.0047 |
|  | BSP | 0.0201 | 0.0083 |
|  |  |  |  |
| yidP | GMS | 0.0153 | 0.0109 |
|  | BSP | 0.0307 | 0.0211 |
|  |  |  |  |
| treR | GMS | 0.0120 | 0.0086 |
|  | BSP | 0.0235 | 0.0144 |
|  |  |  |  |
| Z2975 | GMS | 0.0174 | 0.0136 |
|  | BSP | 0.0358 | 0.0230 |
| Z1440 | GMS | 0.0018 | 0.0302 |
|  | BSP | 0.0031 | 0.0527 |
| Z4981 | GMS | 0.0035 | 0.0069 |
|  | BSP | 0.0056 | 0.0084 |

1. GMS: Genome methylation sequencing

Table S5. Strains and plasmids used in this study

| Strains/plasmids | Description | Source |
| --- | --- | --- |
| EDL933 | ATCC strain | ATCC |
| EDL933ΔmraW | *mraW* gene replace by kan gene from pRS551 in EDL933 | This study |
| DH5α | Chemical competent cell | TaKaRa |
| pRS551 | A 1696 bp *Pvu*II fragment containing the kan gene and promoter from Tn903 inserted between bla and Tl4 in pRS415 | (Oka, et al., 1981) |
| pKOBEG | A thermosensitive replicon that carries the λ phage *redγ*βα operon expressed under the control of the arabinose-inducible pbad promoter | (Datsenko and Wanner, 2000) |
| pBAD/*Myc*-His A | Arabinose inducible expression vector for His tagged fusion proteins | Invitrogen |
| pBADmraW | A 942 bp mraW fragment cloned into pBAD/*Myc*-His A between *Nco*I and *Hind*III sites | This study |
| pGEN-*luxCDABE* | *luxCDABE* cloned into pGEN222 with a constitutive promoter *em7* | Reference |
| pDS17039 | *fliK* promoter (500bp) cloned into pUC19-c | This study |
| pDS17040 | *fhiA* promoter (500bp) cloned into pUC19-c | This study |
| pDS17041 | *fliJ* gene cloned into pUC19-c | This study |

Table S5. Strains and plasmids used in this study (continued)

| Strains/plasmids | Description | Source |
| --- | --- | --- |
| pDS17042 | A 360 bp of *fliR* from 5' end cloned into pUC19-c | This study |
| pDS17043 | A 426 bp of *fliR* from 3' end cloned into pUC19-c | This study |

| Table S6. Primers and oligonucleotide   \| Primer name \| Sequence (5’-3’)^$^ \| Source/Target \| \| --- \| --- \| --- \| \| P1 \| atgatggaaaactataaacatactacggtgctgctggatgaagccgtta \| ( Oka, et al., 1981) \| \| P2 \| gcggctgactgcccacgggttgggtccatacgcatgtccagcggaccatc \| ( Oka, et al., 1981) \| \| P3 \| ctcacgtctgatcctctcgca \| mraW \| \| P4 \| ctgcggtttgtagccattcag \| mraW flanking \| \| P5 \| ccgggctgacaaaagaagtga \| mraW \| \| P6 \| cggtcagtaaacgggtatggt \| mraW \| \| 5-mraWF \| aaaaa**ccatgg**atgatggaaaactataaacatact \| mraW \| \| 3-mraWR \| ggggggg**aagctt**tcatgcgttcgtcctctctg \| mraW \| \| fhiAP1F \| gttgttgaagaagaaattygt \| *fhiA* \| \| fhiAP1R \| ttatttttataaaccatcrcatc \| *fhiA* \| \| fhiAP2F ttagggtaaagygggtagaaatt *fhiA*  ttagggtaaagygggtagaaatt  *fhiA* \| \| \| \| fhiAP2R actaattaccaactcrccaaac *fhiA*  actaattaccaactcrccaaac  *fhiA* \| \| \| \| fhiAP3F \| tygatgygatggtttataaa \| *fhiA* \| \| fhiAP3R \| tacaccactttatccccatacta \| *fhiA* \| \| fliJP1F \| ggygattttgaaagatttgg \| *fliJ* \| \| fliJP1R \| aactattcaaaacaatatcaactttc \| *fliJ* \| \| fliJP2F \| tggagggttatttgtaataagg \| *fliJ* \| \| fliJP2R \| ccaacrattactaatcatcc \| *fliJ* \| \| fliJP3F \| gaaagttgatattgttttgaatagttg \| *fliJ* \| \| fliJP3R \| caacrcraaaaaatcttataca \| *fliJ* \| \| fliKP1F \| tttttttaggggttggag \| *fliK* \| \| fliKP1R \| taccaaacctacaaacrttattt \| *fliK* \| \| fliKP2F aattagtggaygtagaaagttgatat *fliK*  aa \| \| \|   Table S6. Primers used in this study (continued) | | |
| --- | --- | --- | --- | --- | --- | --- | --- | --- | --- | --- | --- | --- | --- | --- | --- | --- | --- | --- | --- | --- | --- | --- | --- | --- | --- | --- | --- | --- | --- | --- | --- | --- | --- | --- | --- | --- | --- | --- | --- | --- | --- | --- | --- | --- | --- | --- | --- | --- | --- | --- | --- | --- | --- | --- | --- | --- | --- | --- | --- | --- | --- | --- | --- | --- | --- | --- | --- | --- | --- | --- | --- | --- | --- | --- |
| Primer name | Sequence (5’-3’)^$^ | Source/Target |
| fliKP2R | tcttatacaacatcrctaactttacc | *fliK* |
| fliRP1F | ataatggttattttggttaagtttgta | *fliR* |
| fliRP1R | aaataacaaccccatttacaaa | *fliR* |
| fliRP2F | tgtaaatggggttgttatttg | *fliR* |
| fliRP2R | taccaaattcaatatcaacaacaa | *fliR* |
| fliRP3F | gatgtttaatttgttgttggat | *fliR* |
| fliRP3R | acaaaaacatcrttaacaaataa | *fliR* |
| fliRP4F | ttgttgttgatattgaatttggtat | *fliR* |
| fliRP4R | tttcraataatccttaaaataacat | *fliR* |
| Z2975P1F | tatygttatttattyggggtgt | *Z2975* |
| Z2975P1R | tcattactctaacttccttcaataac | *Z2975* |
| Z2975P2F | ttattgaaggaagttagagtaatgagt | *Z2975* |
| Z2975P2R | ctttactaattcaaaaaaaaatcaa | *Z2975* |
| Z2975P3F | gatttttttttgaattagtaaagaaat | *Z2975* |
| Z2975P3R | aattctcaacaccraaaaac | *Z2975* |
| yidP1F | gagggttttaatttyggttattat | *yidP* |
| yidP1R | ccatcttactacaaaacccratat | *yidP* |
| yidP2F | tgtttagtaaggttaatgggtaaa | *yidP* |
| yidP2R | ataccaatactcacaaaaatctacttt | *yidP* |
| treR1F | ggattttttttattatttgyg | *treR* |
| treR1R | aaaaatttctttaaacataaaaaatcc | *treR* |
| treR2F | gaggttatatggaatttgatttta | *treR* |
| treR2R | aataaatcacrataaaccaaac | *treR* |
| fhiA RT F | ggattgatcaaccaggcacag | *fhiA* |
| Table S6. Primers used in this study (continued) | | |
| Primer name | Sequence (5’-3’)^$^ | Source/Target |
| fliJ RT F | gaccctgaaagatctggcaga | *fliJ* |
| fliJ RT R | gttgctggcgatgctgagta | *fliJ* |
| fliK RT F | gcaacagataagcccacgaca | *fliK* |
| fliK RT R | gcggtgacgtcttcattcaga | *fliK* |
| fliR RT F | gcgaacaatggctatcctggt | *fliR* |
| fliR RT R | gcgaacaatggctatcctggt | *fliR* |
| crl RT F | cgtactaggcccgtatattcgtg | *crl* |
| crl RT R | gctccagtcgttcaaccacttca | *crl* |
| barA RT F | cagcattattgagccgcttgcag | *barA* |
| barA RT R | gatcataatatcgccgtcacgagt | *barA* |
| recD RT F | cggtataaaccagctctcgcgtt | *recD* |
| recD RT R | gatgattgcccgtaatgacagcg | *recD* |
| mraZ RT F | cgttagtcaatctcgacagcaaa | *mraZ* |
| mraZ RT R | ctaacagtaggcgctgcacac | *mraZ* |
| escD RT F | cgggaattacaacttccgatggg | *escD* |
| escD RT R | ccaatgatgaagcaacatcccgc | *escD* |
| z4187 RT F | gtagccggatccaaagtactactt | *z4187* |
| z4187 RT R | cgccagtgggaatataccaacag | *z4187* |
| espB RT F | ccagattcccctgctggaagttt | *espB* |
| espB RT R | gcgaaagcctctgactttgctga | *espB* |
| htrC RT F | cggatggcgacgttcgtgattta | *htrC* |
| htrC RT R | gtgtgcgcgctaagttataacgg | *htrC* |
| ddg RT F | gccgaaaactttcgttcactcgg | *ddg* |
| ddg RT R | ctgcacccattccatcagctgat | *ddg* |
| grpE RT F | cgaatcgtacgaccattcagcgt | *grpE* |
| Table S6. Primers used in this study (continued) | | |
| Primer name | Sequence (5’-3’)^$^ | Source/Target |
| grpE RT R | gtggctgataaagctaacccgga | *grpE* |
| cspA RT F | gcacagaatctaagatccctgcc | *cspA* |
| cspA RT R | ggagttatgagctggcggcattt | *cspA* |
| Random DNA  sequence | ttcctgttcggtatcccattccaatatagtacctccgccgaactacacaatatatgatggattggctaaatttagcagcaaactttcctggtcaaaatacagaaccgaatacctagaataccactacgaactaaagccgttactggattg | randomly  generated |

^$^Bold sequences are restriction sites. Where Y=C & T, R=A & G.
